# Pharmacogenetic Predictors of Chemotherapy Treatment-Related Toxicities in Paediatric and Adolescent Acute Lymphoblastic Leukemia: A Systematic Review, Meta-Analysis and Literature-Based Candidate Prioritization

**DOI:** 10.3390/ph19081204

**Published:** 2026-08-01

**Authors:** Santenna Chenchula, Suresh Kumar Srinivasamurthy, Vinnyfred Vincent, Himani Thakkar, Shuvadeep Ganguly, Smita Kayal, Swaminathan Keerthivasagam, Jaikumar Ramamoorthy, Swetambri Sharma, Kamali Murugadoss, Archna Singh, Deepam Pushpam, Jayanthi Mathaiyan, Bani Jolly, Vinod Scaria, Yvonne Gloor, Frederic Baleydier, Sameer Bakhshi, Biswajit Dubashi, Marc Ansari, Chakradhara Rao S. Uppugunduri

**Affiliations:** 1 Department of Pharmacology, All India Institute of Medical Sciences (AIIMS), Bhopal 462020, India; csanten7@gmail.com; 2 Department of Pharmacology, RAK College of Medical Sciences, RAK Medical & Health Sciences University, Ras Al Khaimah 11172, United Arab Emirates; sureskums@gmail.com; 3 Division of Endocrinology and Metabolism, Department of Internal Medicine, Carver College of Medicine, Iowa City, IA 52242, USA; vinnyfred-vincent@uiowa.edu (V.V.); himani-thakkar@uiowa.edu (H.T.); 4 Department of Medical Oncology, Jawaharlal Institute of Postgraduate Medical Education and Research (JIPMER), Puducherry 605006, India; ganguly.shuvadeep@gmail.com (S.G.); kayalsmita@gmail.com (S.K.); swami5590@gmail.com (S.K.); kamali.mpgxindall@gmail.com (K.M.); drbiswajitdm@gmail.com (B.D.); 5 Department of Pediatrics, Jawaharlal Institute of Postgraduate Medical Education and Research (JIPMER), Puducherry 605006, India; jaikumarramamoorthy@gmail.com; 6 Department of Medical Oncology, All India Institute of Medical Sciences (AIIMS), New Delhi 110029, India; sswetambri@gmail.com (S.S.); deepampushpam@gmail.com (D.P.); sambakh@hotmail.com (S.B.); 7 Department of Biochemistry, All India Institute of Medical Sciences (AIIMS), New Delhi 110029, India; arch2574@gmail.com; 8 Department of Pharmacology, Jawaharlal Institute of Postgraduate Medical Education and Research (JIPMER), Puducherry 605006, India; drjayanthi2008@gmail.com; 9 Karkinos Healthcare Pvt. Ltd., Mumbai 400021, India; 10 Department of Pediatrics, Gynecology and Obstetrics, CANSEARCH Research Platform in Pediatric Oncology and Haematology, University of Geneva, 1205 Geneva, Switzerland; yvonne.gloor@unige.ch (Y.G.); frederic.baleydier@hug.ch (F.B.); marc.ansari@hug.ch (M.A.)

**Keywords:** acute lymphoblastic leukemia, pharmacogenetics, pharmacogenomics, treatment-related toxicities, children, adolescents, candidate genes, variants

## Abstract

**Background**: Childhood and adolescent acute lymphoblastic leukemia (ALL) survival outcomes have improved significantly, but treatment-related toxicities (TRTs) remain a major concern affecting dose intensity and quality of life. Germline pharmacogenetic variations contribute to inter-individual differences in chemotherapy response, yet consistent replication of associations has been limited by differences in treatment protocols, ethnic backgrounds, and toxicity definitions. **Methods**: This systematic review and meta-analysis (PROSPERO CRD42021229748) included 68 studies in the qualitative synthesis, with 42 high-quality studies undergoing structured synthesis and, where appropriate, quantitative meta-analysis. Studies focused on the toxicities of thiopurines, methotrexate, glucocorticoids, vincristine, and asparaginase. **Results**: Meta-analyses showed strong evidence linking the *NUDT15* rs116855232 variant to thiopurine-induced myelosuppression (OR 19.0, 95% CI 1.6–224; I^2^ = 86.5%) and a significant association between the *TYMS* enhancer repeat polymorphism and osteonecrosis (OR 6.66, 95% CI 3.67–12.11; I^2^ = 0%). In contrast, *MTHFR* variants and *VDR* polymorphisms showed no significant associations with methotrexate-induced myelosuppression or osteonecrosis, respectively. Narrative synthesis highlighted clinically actionable associations: *TPMT* and *NUDT15* with thiopurine toxicity; *CEP72* with vincristine neuropathy; and *HLA* haplotypes with asparaginase hypersensitivity. Population allele frequency comparisons (IndiGenomes, gnomAD, UK Biobank) showed strong concordance (r = 0.919–0.997), supporting the broad generalisability of identified variants, which remains to be evaluated prospectively. The main limitations included heterogeneous toxicity definitions, non-uniform genetic models, evolving treatment protocols, population heterogeneity, and a lack of harmonised reporting. **Conclusions**: The review supports routine *TPMT* and *NUDT15* genotyping and highlights the need for harmonised definitions, multi-ethnic studies, standardised data representation, and prospective validation. A literature-based candidate gene list for future PGx association studies was generated.

## 1. Introduction

The survival outcomes of childhood acute lymphoblastic leukemia (ALL) have improved remarkably over the past few decades and are widely regarded as one of the major success stories of modern oncology. This progress has largely been driven by the uniform adoption of risk-stratified, protocol-based, multi-agent chemotherapy regimens [1]. Standard treatment protocols for childhood ALL typically include antimetabolites (thiopurines, methotrexate, and cytarabine), glucocorticoids (prednisolone and dexamethasone), microtubule inhibitors (vincristine), and alkylating agents (daunorubicin and cyclophosphamide) [2]. Despite their therapeutic efficacy, these agents are frequently associated with treatment-related toxicities (TRTs), including myelosuppression (with consequent risk of severe infection), peripheral neuropathy, pancreatitis, thrombosis, and hepatotoxicity. Such toxicities can adversely affect treatment outcomes and substantially impair quality of life during and after therapy [3].

Although significant advances have been made in understanding the biology of ALL and mechanisms of drug resistance (which are crucial for treatment efficacy), the ability to predict, prevent, or mitigate TRTs, which largely depend on drug mechanisms of action and host pharmacogenetics, remains limited to some extent [4]. In this context, germline pharmacogenetic variations have emerged as important contributors to inter-individual differences in drug response and toxicity. Several studies have investigated associations between germline variants in genes encoding drug-metabolizing enzymes, transporters, receptors, and other pharmacological targets and the risk of TRTs in children and adolescents receiving chemotherapy for ALL [5]. These genetic variants may influence drug disposition and sensitivity, potentially leading to increased susceptibility to toxicity and, in some cases, an adverse prognostic impact [5].

Importantly, the clinical relevance of some pharmacogenetic variation is supported by contemporary international guidelines. The Clinical Pharmacogenetics Implementation Consortium (CPIC) recommends genotype-guided dosing of thiopurines based on *TPMT* and *NUDT15* variants to reduce the risk of severe myelosuppression, with recent updates emphasising combined genotype effects and phenotype-based dose adjustments [6]. Similarly, the Dutch Pharmacogenetics Working Group (DPWG) classifies *TPMT* and *NUDT15* genotyping as essential prior to thiopurine initiation and provides evidence-based dosing recommendations to mitigate toxicity risk [7]. These guideline-driven approaches highlight the growing clinical utility of pharmacogenomics (PGx) and support its integration into treatment individualisation in paediatric ALL.

However, the implementation, interpretation and clinical translation of many other findings remain challenging. Reported associations between genetic variants and TRTs are not consistently reproducible across studies, likely due to differences in treatment protocols, study design, outcome definitions, and ethnic backgrounds of the populations studied [8]. Moreover, it remains controversial whether specific germline variants associated with TRTs also translate into poorer relapse-free survival [9,10]. Consequently, the causal relevance and clinical utility of many reported PGx associations are still uncertain.

Despite the growing body of literature evaluating germline genetic determinants of TRTs across different phases of ALL treatment, a systematic and comprehensive synthesis of this evidence, particularly with respect to rigorous quality assessment of included studies, is limited. Such a synthesis is essential to clarify the strength and consistency of reported associations and to develop pharmacogenetics-guided treatment strategies in childhood ALL. In addition, identifying and prioritizing clinically relevant candidate genes and variants based on existing evidence will aid in selecting candidates for future prospective PGx association studies, including ongoing observational initiatives such as MPGx-INDALL [11].

Therefore, in this systematic review and meta-analysis, we aimed to comprehensively evaluate the available evidence on the associations between germline pharmacogenetic variants and TRTs in children and adolescents with ALL, across a broad range of toxicities, genetic variants, and ethnic groups. Specifically, we sought to identify high-risk genetic variants associated with an increased incidence of TRTs, taking into consideration the quality aspect of the study report and, where feasible, to quantify the magnitude of these associations by estimating pooled effect measures, including odds ratios, relative risks, hazard ratios, or cumulative incidence, comparing carriers of risk alleles with non-carriers.

## 2. Methods

### 2.1. Eligibility Criteria

The study protocol was registered with PROSPERO (International Prospective Register of Systematic Reviews; CRD42021229748) and conducted in accordance with the Preferred Reporting Items for Systematic Reviews and Meta-Analyses (PRISMA) guidelines [12]. We included observational comparative studies (prospective and retrospective cohorts), randomized controlled trials, and cross-sectional studies that evaluated associations between germline pharmacogenetic variants and the development of treatment-related toxicities (TRTs) during chemotherapy in pediatric and adolescent patients (≤21 years) with de novo ALL.

We excluded reviews, case reports, case series, editorials, personal opinions, book chapters, clinical guidelines, and conference abstracts. Studies involving relapsed ALL or patients treated in the setting of hematopoietic stem-cell transplantation were also excluded. Eligible studies were required to compare one or more germline pharmacogenetic risk variants with an appropriate comparator group and to report summary statistics describing the association with TRTs. Genetic variants without established pharmacogenetic relevance and variants associated only with a general predisposition to toxicity, without drug-specific implications, were not considered. Studies reporting PGx associations in mixed-age populations (pediatric and adult) were excluded unless pediatric data could be clearly disaggregated.

The primary outcomes of interest were either the proportion or incidence of the following TRTs occurring during any phase of ALL treatment: myelosuppression (anaemia, leucopenia, and thrombocytopenia; grades 3–5), neuropathy (all grades), hepatotoxicity (grades 3–5), nephrotoxicity (grades 3–5), pancreatitis (all grades), thrombosis or thromboembolic events (all grades), hyperglycaemia (all grades), allergic reactions or hypersensitivity (grades 3–5), and osteonecrosis (all grades). Only studies reporting TRTs defined according to the National Cancer Institute Common Terminology Criteria for Adverse Events (NCI-CTCAE; all versions) were included [13]. Secondary outcomes included disease-free survival, relapse-free survival, and overall survival. Studies reporting only secondary outcomes without data on TRTs were excluded.

### 2.2. Search Strategy and Study Selection

We systematically searched PubMed, Embase, Scopus, and Web of Science using database-specific strategies (Supplementary Material S1). We included English-language studies published between 10 January 1995 and 10 January 2021. To support validation of findings (e.g., population allele frequency comparisons), we extended the search to March 2026; however, this extended search was used only to verify associations confirmed from earlier search, and no additional studies were included in the primary review or meta-analysis.

Study selection was managed using the open-access CADIMA tool [14]. Duplicate records were removed using EndNote X9 (Clarivate Analytics, Philadelphia, PA, USA). Titles and abstracts were independently screened by two reviewers, followed by full-text examination. Discrepancies were resolved through discussion and, if needed, adjudication by a third reviewer. The detailed protocol and extracted datasets are available through the CADIMA platform (Supplementary Material S2).

### 2.3. Data Extraction

Data were extracted using a predesigned spreadsheet template (Supplementary Material S3). Extracted variables included study characteristics, participant demographics, treatment protocols, genetic variants, genotyping methods, toxicity outcomes, and effect measures. Effect measures included proportions, odds ratios, relative risks, cumulative incidences, hazard ratios, or survival estimates. Automated data extraction using AI tools was also attempted.

### 2.4. Quality Assessment

Study quality was assessed using an adapted version of the STrengthening the Reporting of Pharmacogenetic Studies (STROPS) checklist [15]. From the original 54-item checklist, 23 items most relevant to pharmacogenetic association studies were selected by consensus among reviewers. These items assess participant selection, genotyping validity, outcome definitions, statistical analyses, and reporting transparency. The full list of included and excluded items, along with the rationale for exclusion and the consensus process, is provided in Supplementary Material S4.

Each item was scored as 1 if fulfilled, 0 if not reported. Two reviewers independently assessed each study, with disagreements resolved by a third reviewer. Studies were categorised as low (0–7), moderate (8–15), or high quality (16–23) based on predefined thresholds. Studies with scores below the lower limit were excluded from quantitative meta-analysis but retained in the qualitative synthesis.

### 2.5. Data Synthesis and Statistical Analysis

A qualitative synthesis summarised associations between germline variants and each TRT, documenting the drug, chemotherapy phase, genetic model, and ethnicity where available. Meta-analysis was performed when at least two independent studies reported comparable genetic variants and toxicity outcomes.

For genotype-specific data, 2 × 2 contingency tables were constructed from toxicity events among risk-genotype and non-risk-genotype carriers. Study-specific odds ratios (ORs) with 95% confidence intervals (CIs) were calculated when not directly reported. Pooled estimates were calculated using a random-effects model with the Mantel–Haenszel method [16,17]. For studies reporting only summary estimates, the generic inverse-variance method was used [18].

Heterogeneity was assessed using Cochran’s Q test and quantified with the I^2^ statistic (low: 25%, moderate: 50%, high: 75%) [19]. Between-study variance (τ^2^) was estimated using restricted maximum likelihood (REML) [18]. Forest plots were generated using R (meta and metafor packages) [20,21]. Statistical significance was set at *p* < 0.05. Sensitivity analyses used a leave-one-out approach [22]. Publication bias was assessed by funnel plot inspection and Egger’s linear regression test [23,24].

### 2.6. Population Allele Frequency Comparison

Minor allele frequencies (MAFs) of PGx variants identified in the systematic review were compared across population datasets to assess their generalizability. Variants were mapped to corresponding rsIDs in the Indigenome dataset [25], and MAFs were extracted for comparison with the Genome Aggregation Database (gnomAD) populations, including all populations (AF), South Asian (SAS), and non-Finnish European (NFE) groups [26]. A Pearson correlation analysis was performed to assess the concordance between allele frequencies observed in the Indigenome and gnomAD populations [27]. Scatter plots were generated to visualize the relationships between allele frequencies across populations. A Fisher’s exact test with Bonferroni correction was used to evaluate statistically significant differences in allele frequencies between populations [28].

Additionally, allele frequencies of identified variants were compared between individuals of Indian ancestry and British ancestry in the UK Biobank dataset (Application ID: 91515) [29] to assess population consistency. Linear regression analysis was used to evaluate correlations between allele frequencies across these populations [27].

## 3. Results

### 3.1. Study Selection

The systematic literature search identified 2024 records across PubMed, Embase, Scopus, and Web of Science. After removing 219 duplicate records, 1805 studies underwent title and abstract screening. Of these, 214 full-text articles were assessed for eligibility. A total of 146 studies were excluded, primarily due to an ineligible study population or absence of pharmacogenetic variants associated with treatment-related toxicities (TRTs) (n = 32), reporting of late or delayed toxicities without standardized NCI-CTCAE grading definitions (n = 105), or ineligible publication type (n = 9).

**Figure 1:**
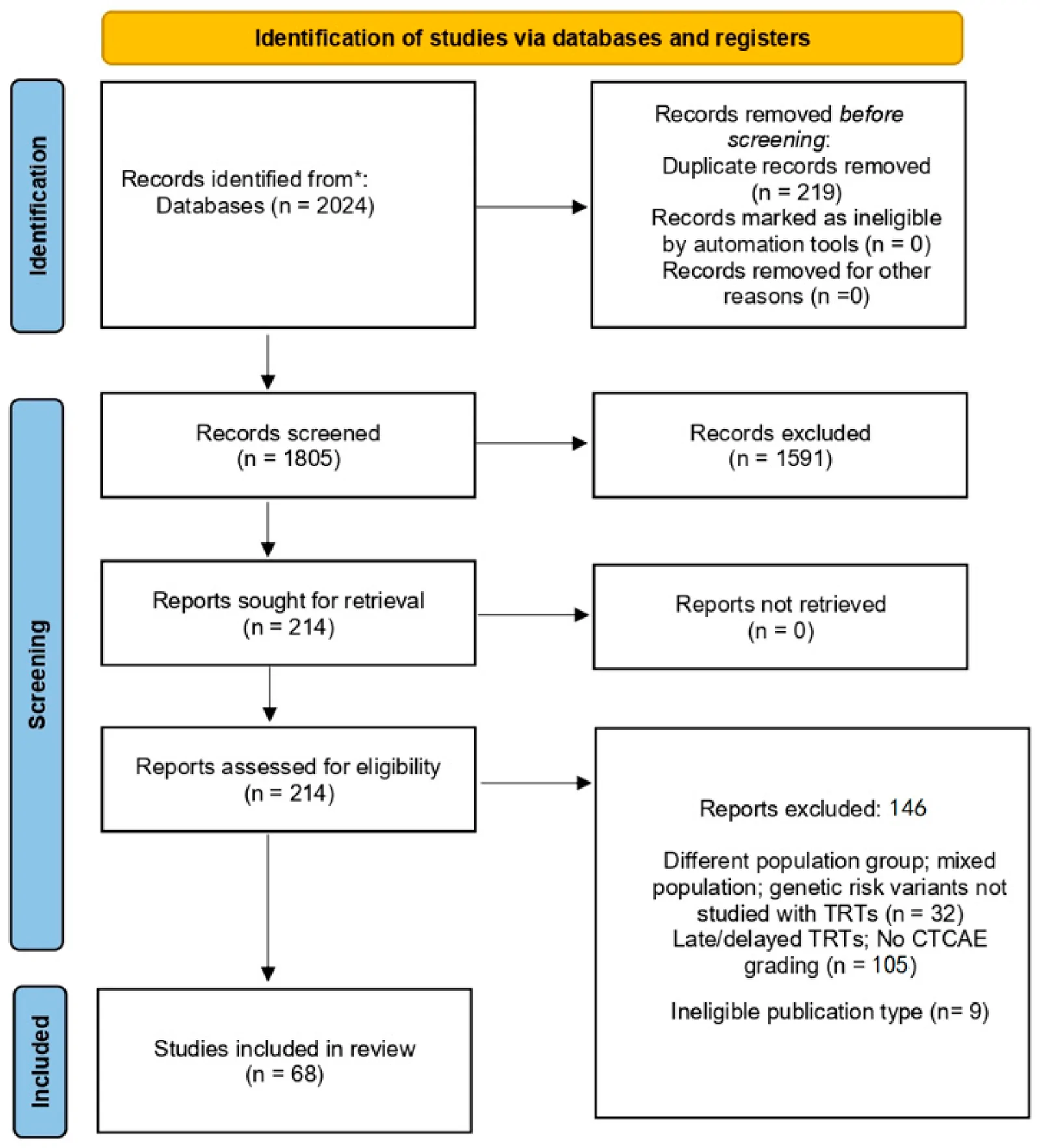
PRISMA 2020 flow diagram illustrating the study selection process for the systematic review and meta-analysis. * Note: Records were identified from multiple sources, including databases (PubMed, Embase, Scopus, and Web of Science).

Ultimately, 68 studies fulfilled the inclusion criteria and were included in the qualitative synthesis [30,31,32,33,34,35,36,37,38,39,40,41,42,43,44,45,46,47,48,49,50,51,52,53,54,55,56,57,58,59,60,61,62,63,64,65,66,67,68,69,70,71,72,73,74,75,76,77,78,79,80,81,82,83,84,85,86,87,88,89,90,91,92,93,94,95,96,97]. Detailed characteristics of the included studies are presented in Supplementary Table S1. Among these, 42 studies met predefined high-quality criteria based on the adapted STROPS assessment (score ≥ 16) and were therefore eligible for structured synthesis and, where appropriate, quantitative meta-analysis [30,31,32,33,34,35,36,37,38,39,40,41,42,43,44,45,46,47,48,49,50,51,52,53,54,55,56,57,58,59,60,61,62,63,64,65,66,67,68,69,70,71]. The study selection process is summarised in Figure 1.

### 3.2. Study Characteristics

The 68 included studies comprised prospective and retrospective cohort studies, genome-wide association studies (GWAS), and PGx analyses embedded within cooperative clinical trial protocols [30,31,32,33,34,35,36,37,38,39,40,41,42,43,44,45,46,47,48,49,50,51,52,53,54,55,56,57,58,59,60,61,62,63,64,65,66,67,68,69,70,71,72,73,74,75,76,77,78,79,80,81,82,83,84,85,86,87,88,89,90,91,92,93,94,95,96,97]. Sample sizes ranged from 30 to 2285 pediatric patients with de novo ALL.

The included studies represented diverse ethnic populations, including East Asian cohorts [30,37,46,61,80,86,87], European populations [32,34,43,47,49,55,56,64,70], North American cohorts [44,48,55,58,65], Middle Eastern populations [49,62], South Asian populations [53,82], and Hispanic cohorts [47,78].

Treatment protocols largely followed established cooperative group regimens, including those developed by the Children’s Oncology Group (COG), Dana-Farber Cancer Institute (DFCI), Berlin–Frankfurt–Münster (BFM), and the Nordic Society of Pediatric Hematology and Oncology (NOPHO), along with related national adaptations [32,36,43,44,47,54]. However, variability in glucocorticoid exposure, chemotherapy dosing intensity, and supportive care practices was observed across studies.

The most frequently investigated pharmacogenetic pathways included thiopurine metabolism genes (*TPMT*, *NUDT15*, *ITPA*, *IMPDH1*) [30,48,49,53,66,67,80,84,85,86,96,97]; folate and methotrexate pathway genes (*MTHFR*, *TYMS*, *DHFR*, *SLCO1B1*, *ABCC2*, *ABCB1*) [30,31,37,38,39,42,46,61,74,76,77,80,83,85,87,92,94]; asparaginase associated toxicity genes (*ULK2*, *RGS6*, *HLA*, *GRIA1*, *ASNS*) [36,43,45,47,50,60,71,78]; glucocorticoid associated osteonecrosis genes (*ACP1*, *SERPINE1*, *BCL2L11*, *GRIN3A*, *ESR1*) [32,34,44,49,56,57,63,64,70]; and vincristine induced neuropathy genes (*CEP72*, *ACTG1*, *ABCB1*, *SYNE2*) [41,52,55,95]. Detailed characteristics of the 42 high-quality studies are summarised in Table 1.

Among the 68 included studies, the most frequently represented ethnic regions were the Americas (AMR, 23 studies, 33.8%), Europe (16 studies, 23.5%), and East Asia (16 studies, 23.5%); South Asian, African, and Middle Eastern cohorts each accounted for less than 8% of studies. Regarding genetic pathways, folate/methotrexate pathway genes were investigated most often (28 studies, 41.2%), followed by thiopurine metabolism genes (19 studies, 27.9%); a further 10 studies (14.7%) examined both. The majority of studies (42 studies, 61.8%) met high-quality criteria (STROPS score ≥ 16). Described genetic associations with the treatment related adverse effects are depicted in Table 2.

### 3.3. Data Verification

Data verification was performed by a collaborator who was not involved in the data extraction. Extracted data were independently verified using automated data-checking tools (Deben Services Ltd., Suffolk, UK). As automated verification was not feasible for complex SNP-level data, these data were manually cross-checked against the original publications by an independent review team to ensure accuracy and consistency. Auto verification using AI tools and a script was not successful owing to the heterogeneity in the presentation of the data and the way the analysis results were presented in the reports.

### 3.4. Quality Assessment

**Figure 2:**
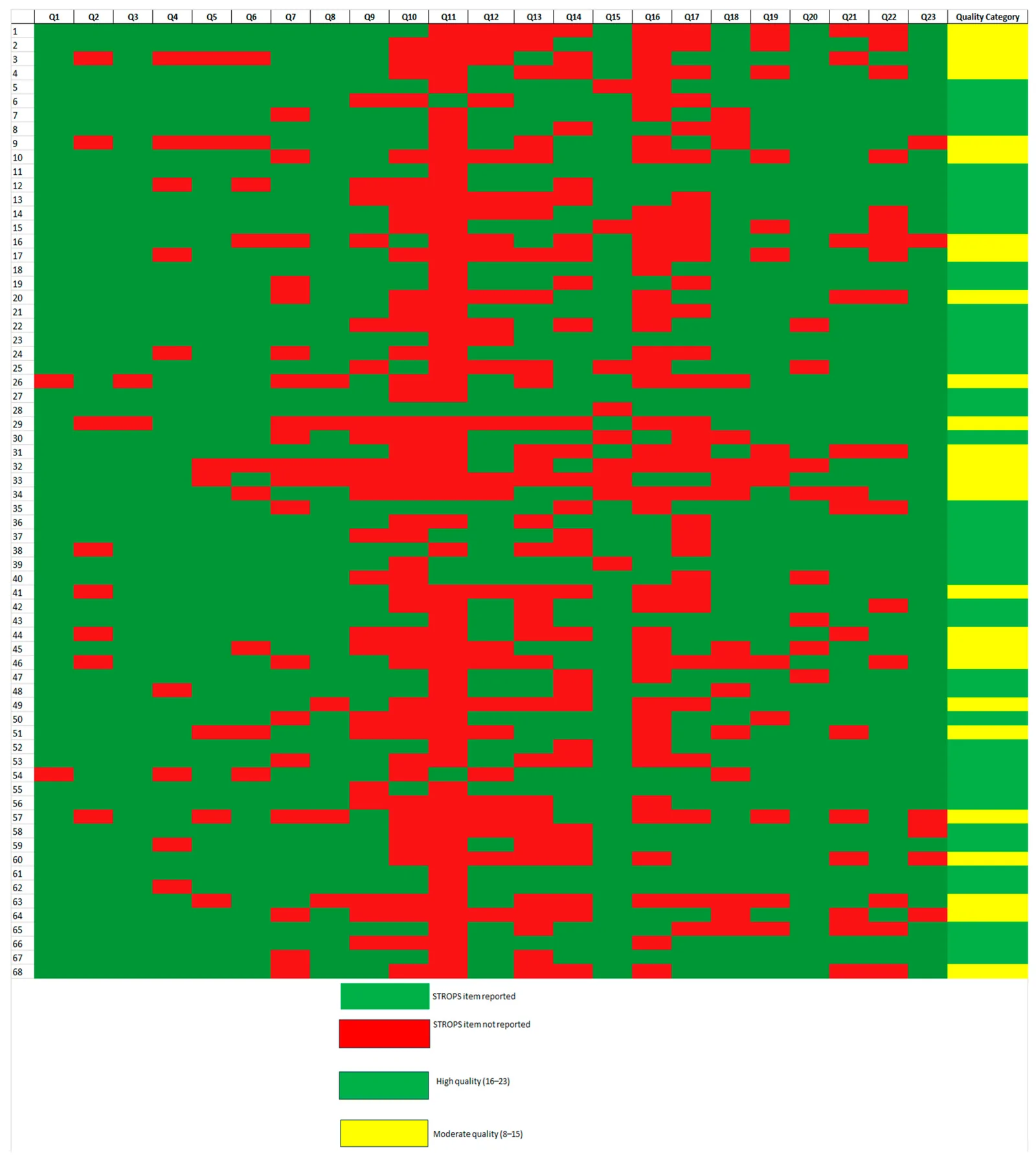
Heatmap showing compliance with the 23-item STROPS (Strengthening the Reporting of Pharmacogenetic Studies) checklist across the 68 included studies. Each row represents an individual study, and each column represents a STROPS reporting item (Q1–Q23) (See Supplementary Material S4 for details of questions). Green cells indicate that the reporting criterion was fulfilled, whereas red cells indicate that the criterion was not reported. The final column indicates the overall study quality based on the total STROPS score, with green representing high-quality studies (scores 16–23) and yellow representing moderate-quality studies (scores 8–15).

The distribution of STROPS checklist items across studies is illustrated in Figure 2, and detailed item-level scoring is provided in Supplementary Table S2 and Supplementary Figure S1.

Common methodological limitations among moderate-quality studies included small sample sizes [73,90], incomplete reporting of Hardy–Weinberg equilibrium testing [58,81], lack of multivariable adjustment for potential confounders [88], and inconsistent toxicity grading criteria [94]. Not applying multiple testing correction was another limitation noted in many reports. In contrast, high-quality studies more consistently reported validated genotyping methods, genotype frequencies, predefined genetic models, and toxicity outcomes defined according to NCI-CTCAE criteria [32,34,36,55,56,64]. The most missed STROPS items among moderate-quality studies included inadequate reporting of Hardy–Weinberg equilibrium, lack of multivariable adjustment for potential confounders, and incomplete specification of genetic models.

### 3.5. Quantitative Synthesis

Quantitative meta-analysis was performed for osteonecrosis (three genetic variants) and for selected single variants associated with myelosuppression (*NUDT15* rs116855232 for thiopurine toxicity and *MTHFR* c.677C>T for methotrexate toxicity).

### 3.6. Genetic Variants and Osteonecrosis

**Figure 3:**
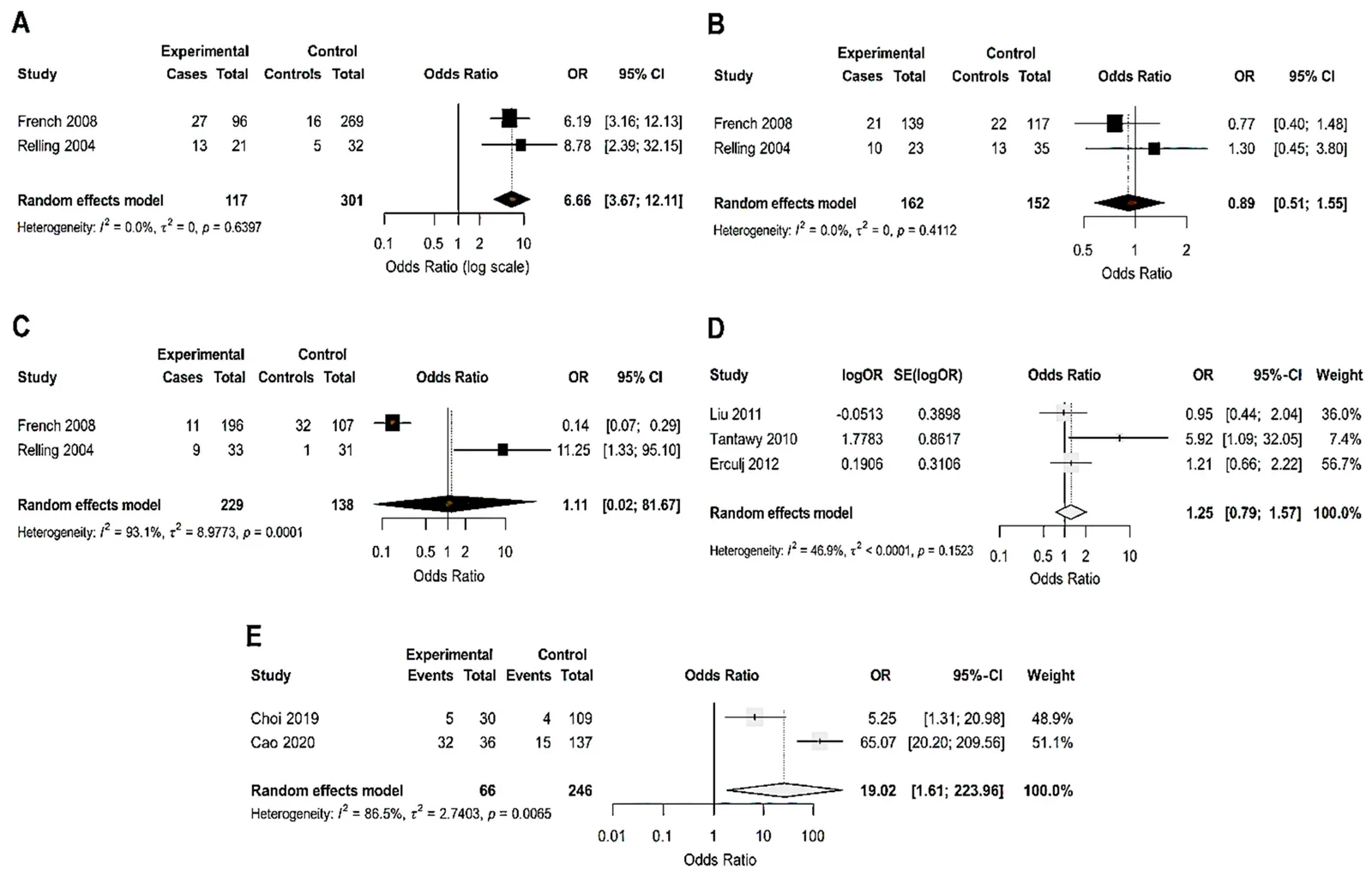
Forest plots of meta-analyses for genetic variants associated with treatment-related toxicities in paediatric ALL. (**A**) *TYMS* enhancer repeat polymorphism (risk genotype: carriers of at least one 3R allele) and osteonecrosis [32,44]. (**B**) *MTHFR* rs1801133 (risk allele: T; reference allele: C) and osteonecrosis [32,44]. (**C**) *VDR* FokI rs2228570 (risk allele: T; reference allele: C) and osteonecrosis [32,44]. (**D**) *MTHFR* c.677C>T (risk allele: T; reference allele: C) and methotrexate-induced myelosuppression (inverse-variance model) [39,61,62]. (**E**) *NUDT15* rs116855232 (risk allele: T; reference allele: C) and thiopurine-induced myelosuppression [30,66]. For panels (**A**–**C**,**E**), the risk genotype group includes patients carrying at least one risk allele (i.e., CT or TT, or the equivalent for the *TYMS* polymorphism), and the non-risk genotype group includes patients homozygous for the reference allele (or the 2R/2R genotype for *TYMS*). Panel (**D**) displays effect estimates (log-odds ratios) with their standard errors extracted from each study. Pooled effect estimates were calculated using random-effects Mantel–Haenszel models (**A**–**C**,**E**) or a random-effects inverse-variance model (**D**). Individual studies are represented by squares proportional to their statistical weight; horizontal lines represent the 95% confidence intervals (CI). Diamonds indicate the pooled odds ratios (OR) with their 95% CIs. Heterogeneity among studies was assessed using the I^2^ statistic.

Variant-specific meta-analyses were conducted for three pharmacogenetic loci: *MTHFR* rs1801133, *TYMS* enhancer repeat polymorphism, and *VDR* FokI (rs2228570). For the *MTHFR* rs1801133 polymorphism, two studies were included [32,44]. The pooled random-effects meta-analysis showed no significant association between the *MTHFR* variant and osteonecrosis risk (OR = 0.89, 95% CI 0.51–1.55; I^2^ = 0%, *p* = 0.41) (Figure 3B).

For the *TYMS* enhancer repeat polymorphism, two independent studies were included [32,44]. These two studies are from independent cohorts, including French et al. (2008) [32], who used patients from the Children’s Cancer Group (CCG) protocols, while Relling et al. (2004) [44] used patients from St. Jude Children’s Research Hospital, with no patient overlap. The pooled random-effects meta-analysis demonstrated a significant association between the risk genotype and osteonecrosis (OR = 6.66, 95% CI: 3.67–12.11), with no evidence of statistical heterogeneity (I^2^ = 0%, *p* = 0.64) (Figure 3A).

For the *VDR* FokI (rs2228570) polymorphism, two studies were included [32,44]. The pooled random-effects meta-analysis did not demonstrate a statistically significant association with osteonecrosis risk (OR = 1.11, 95% CI 0.02–81.67), with substantial heterogeneity between studies (I^2^ = 93.1%, *p* = 0.0001) (Figure 3C).

### 3.7. Genetic Variants and Myelosuppression

For thiopurine-related toxicity, two independent studies evaluating the *NUDT15* rs116855232 variant were available for meta-analysis [30,66]. The pooled random-effects meta-analysis demonstrated a significantly increased risk of myelosuppression among variant carriers (OR = 19.02, 95% CI 1.61–223.96), with substantial heterogeneity between studies (I^2^ = 86.5%, *p* = 0.0065) (Figure 3E).

For methotrexate-related toxicity, three studies evaluating the *MTHFR* C677T polymorphism were included, using a generic inverse-variance model [39,61,62]. The pooled analysis showed no statistically significant association between the variant and myelosuppression risk (OR = 1.25, 95% CI 0.79–1.97), with moderate heterogeneity observed (I^2^ = 46.9%, *p* = 0.152) (Figure 3D).

Overall, because fewer than ten studies were available for each analysis, formal assessment of publication bias was not performed, consistent with methodological recommendations [20,21].

### 3.8. Narrative Synthesis of High-Quality Studies

#### 3.8.1. Thiopurine-Induced Myelosuppression

Strong and reproducible associations were observed between thiopurine-related cytopenias and variants in *TPMT* and *NUDT15*. The *NUDT15* rs116855232 variant was consistently associated with early-onset leukopenia, prolonged cytopenias, and markedly reduced tolerated 6-mercaptopurine doses, particularly in East Asian populations [30,80,84,86,97]. Similarly, *TPMT* polymorphisms were associated with neutropenia, infection risk, dose interruption, and mercaptopurine intolerance across multiple ethnic populations [48,49,53,67,84,85]. Additional modifiers, including *ITPA* and *IMPDH1*, were implicated in several studies [30,53,75], although replication across cohorts was not consistent.

Despite the large number of studies, meta-analysis for *TPMT*, *ITPA*, and *IMPDH1* was not feasible because studies used different genetic models (e.g., dominant vs. recessive), reported diplotypes rather than single variants, or defined myelosuppression using non-uniform cut-offs (e.g., grade 3–4 vs. any grade; leukopenia vs. neutropenia). Overall, thiopurine pharmacogenetics demonstrated the most consistent and clinically actionable evidence among the toxicities evaluated.

#### 3.8.2. Methotrexate-Related Toxicities

Variants in *MTHFR*, *TYMS*, *DHFR*, *SLCO1B1*, *ABCC2*, and *ABCB1* were associated with hepatotoxicity, mucositis, cytopenias, delayed methotrexate clearance, and elevated plasma methotrexate concentrations [30,31,37,38,39,42,46,61,74,76,77,80,83,85,87,92,94]. The *MTHFR* C677T polymorphism was associated with increased hematologic toxicity and altered methotrexate exposure in several cohorts [39,61,69,76,82,85], although findings were inconsistent across populations [80,83]. Variants in *SLCO1B1* were associated with delayed methotrexate clearance and an increased risk of mucositis [46], while *ABCC2* polymorphisms were linked to higher methotrexate plasma concentrations and hematologic toxicity [38,74].

Quantitative synthesis was possible only for *MTHFR* C677T and myelosuppression (Figure 3D). For all other gene-toxicity pairs (e.g., *TYMS* with hepatotoxicity, *ABCC2* with mucositis), meta-analysis was precluded by heterogeneity in outcome definitions (e.g., hepatotoxicity grades 3–5 vs. any elevation of liver enzymes), different phenotyping time points (peak plasma MTX concentration vs. delayed clearance), and inconsistent genetic models (dominant, recessive, or additive). Despite biological plausibility, effect sizes varied considerably across populations.

#### 3.8.3. Asparaginase-Associated Toxicities

Genome-wide and candidate-gene studies identified *ULK2* and *RGS6* variants as significantly associated with asparaginase-associated pancreatitis [43,60]. *HLA* haplotypes, particularly *HLA-DRB1–DQA1–DQB1* combinations, were strongly associated with PEG-asparaginase hypersensitivity [45,54,71]. Variants in *GRIA1* and related pathways were associated with hypersensitivity reactions in selected cohorts [45,50]. *ASNS* pathway polymorphisms demonstrated mixed findings, with some variants conferring increased pancreatitis risk and others appearing protective [50,78].

Meta-analysis could not be performed because the toxicity outcomes were rare (pancreatitis, anaphylaxis), studies used different asparaginase formulations (PEG asparaginase vs. native *E. coli*), and genotype data were often reported as haplotype frequencies rather than as extractable 2 × 2 tables for individual variants. The lack of standardised definitions for hypersensitivity (e.g., NCI-CTCAE grade 3–4 vs. clinical judgment alone) further limited comparability.

### 3.9. Glucocorticoid-Induced Osteonecrosis

Multiple GWAS and candidate-gene studies identified associations between osteonecrosis and variants in *SERPINE1* (PAI-1), *ACP1*, *BCL2L11*, *GRIN3A*, and glucocorticoid receptor-related loci [32,34,44,49,56,57,63,64,70]. Age greater than 10 years consistently emerged as a strong clinical risk factor interacting with genetic susceptibility [44,57,64]. Pathway analyses suggested involvement of glutamate receptor signalling and lipid metabolism pathways [34,63].

Quantitative synthesis was feasible only for three variants (*MTHFR* rs1801133, *TYMS* enhancer repeat, *VDR* FokI), where reporting was sufficiently uniform (Figure 3A–C). For all other reported loci, meta-analysis was prevented by the use of genome-wide significance thresholds that identified single variants not replicated in other cohorts, different definitions of osteonecrosis (symptomatic vs. radiological lesions, hip-specific vs. any joint), and variability in glucocorticoid type (prednisone vs. dexamethasone) and cumulative dose.

### 3.10. Vincristine-Induced Peripheral Neuropathy

The *CEP72* rs924607 TT genotype was significantly associated with increased incidence and severity of vincristine-induced peripheral neuropathy in several independent cohorts [52,55]. Additional loci, including *ACTG1*, *SYNE2*, and *ABCB1* promoter variants, were implicated in neuropathy susceptibility [41,52,87,95], although replication across populations remained variable.

Meta-analysis could not be performed for the other loci because studies used different neuropathy grading scales (NCI-CTCAE vs. paediatric modified total neuropathy scores), assessed neuropathy at different time points (acute vs. persistent), or did not report genotype-specific event counts.

### 3.11. Narrative Synthesis of Moderate-Quality Studies

Moderate-quality studies generally supported the direction of associations observed in high-quality cohorts but were limited by smaller sample sizes and heterogeneity in toxicity definitions [72,88,90]. Reported associations included *ABCB1* variants with infection risk [88], *XRCC1* polymorphisms with myelosuppression [90], *CFTR* variants with pancreatitis [78], and additional *MTHFR* associations with methotrexate-related cytopenias [58,81]. Due to methodological variability, these findings were not quantitatively synthesised.

### 3.12. Population Allele Frequency Comparison

Among the 190 unique variants identified in the systematic review, allele frequency data were available for 130 (68.4%) in the Indigenome dataset, while 60 (31.6%) lacked corresponding entries and were excluded from further population-comparison analysis.

**Figure 4:**
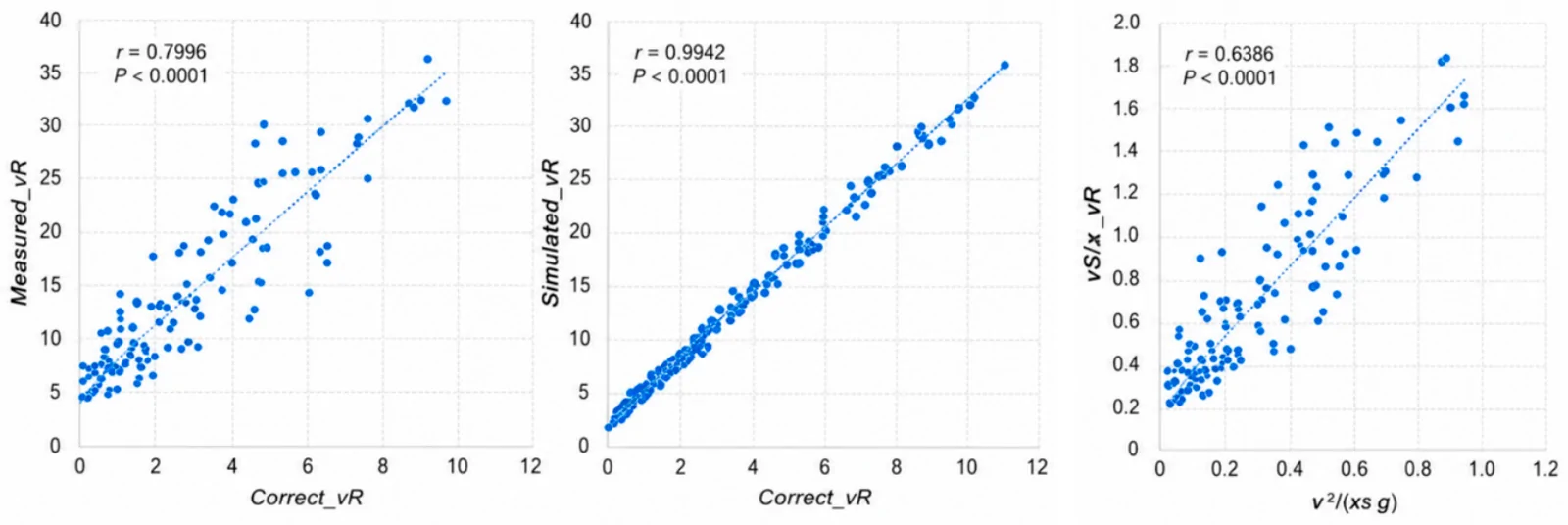
Comparison of minor allele frequencies (MAF) of pharmacogenetic variants between the IndiGenomes dataset and the gnomAD population dataset. Scatter plots illustrate the correlation between allele frequencies observed in the IndiGenomes dataset and those reported in gnomAD populations. Each point represents a pharmacogenetic variant included in the analysis. The diagonal line represents the expected distribution if allele frequencies were identical across datasets. Pearson correlation coefficients were calculated to assess the strength of association between datasets.

Strong correlations were observed between allele frequencies in the Indigenome dataset and those reported in gnomAD populations. Pearson correlation coefficients were 0.919 for all populations (AF), 0.997 for the South Asian population (SAS), and 0.901 for the non-Finnish European population (NFE) (Supplementary Table S3). These results indicate a high degree of concordance in allele frequencies across populations (Figure 4). Fisher’s exact testing identified only a small proportion of variants with significant allele frequency differences after Bonferroni correction. Specifically, 3 variants (2.3%) differed between Indigenome and gnomAD overall populations; none differed in the South Asian population; and 7 variants (5.4%) differed in the non-Finnish European population (Supplementary Figure S2). Comparison of allele frequencies using the UK Biobank dataset demonstrated similar patterns of concordance between Indian and British populations. Linear regression analysis showed a strong relationship between allele frequencies for variants identified in the systematic review (β = 0.907, adjusted R^2^ = 0.799) (Supplementary Figure S3).

## 4. Discussion

This systematic review and meta-analysis synthesize evidence on germline PGx predictors of TRTs in pediatric and adolescent patients with acute lymphoblastic leukemia (ALL). Previous reviews have noted that relatively few pharmacogenetic associations in pediatric ALL toxicity have been consistently replicated across independent cohorts, largely due to differences in treatment protocols, ancestry distributions, and insufficient cohort sizes, which lead to low statistical power [98]. In this systematic review of 68 studies and the variant-level meta-analyses for which data were sufficiently comparable, we identified a few pharmacogenetic associations with strong clinical relevance, while also highlighting the considerable heterogeneity that continues to limit broader clinical implementation in this domain of Pharmacogenomics. Our review is the first in this field to apply the STROPS quality assessment framework, to quantitatively synthesise the association of the TYMS enhancer repeat with osteonecrosis, and to provide a literature-based candidate gene panel for prospective validation. Additionally, we systematically evaluated population allele frequency concordance of selected variants and dissected sources of heterogeneity, offering a roadmap for future research.

The most consistent and clinically actionable evidence emerged for thiopurine metabolism genes, particularly *TPMT* and *NUDT15*. Variants in these genes have demonstrated strong, reproducible associations with thiopurine-induced myelosuppression across multiple populations, including adults with autoimmune diseases and mixed-age cancer cohorts [30,40,41,45,58,59,72,76,77,78]. However, most of these studies were not restricted to pediatric ALL, underscoring the need for dedicated evidence synthesis in this age group. Our quantitative synthesis further confirmed a markedly increased risk of myelosuppression among carriers of the *NUDT15* rs116855232 variant. Although the confidence interval was wide due to the small number of homozygous carriers in the included cohorts, the magnitude and direction of the association are consistent with previous pharmacogenetic studies demonstrating that *NUDT15* variants markedly impair thiopurine metabolism and predispose patients to severe hematopoietic toxicity [6,99]. These findings are further supported by meta-analytic evidence demonstrating strong associations between *NUDT15* variants and thiopurine-induced leukopenia and neutropenia, particularly in Asian populations [99]. Aligning with this evidence, the Clinical Pharmacogenetics Implementation Consortium (CPIC) recommends genotype-guided thiopurine dosing based on *TPMT* and *NUDT15* variants [100]. Recent multiethnic cohort analyses have also demonstrated additive effects of *TPMT* and *NUDT15* variants on thiopurine toxicity, showing that patients with reduced activity in both enzymes can tolerate substantially lower mercaptopurine doses and exhibit increased hematopoietic toxicity compared with carriers of single variants [101]. Pre-emptive identification of loss-of-function alleles in these genes enables substantial dose reduction or alternative therapy to prevent severe thiopurine-induced myelosuppression while maintaining therapeutic efficacy, representing one of the most widely implemented examples of PGx-guided therapy in pediatric oncology [102].

Beyond thiopurine pharmacogenetics, substantial evidence also supports genetic predictors of other drug-specific toxicities. In particular, the *CEP72* rs924607 TT genotype has been consistently associated with an increased risk of vincristine-induced peripheral neuropathy, while specific *HLA* haplotypes, most notably *HLA-DRB1–DQA1–DQB1* combinations, have been linked to PEG-asparaginase hypersensitivity reactions [37,44,46,47,63]. These associations have been replicated across independent cohorts and have plausible biological mechanisms. Functional studies demonstrate that *CEP72* influences neuronal sensitivity to vincristine-induced microtubule disruption, whereas *HLA* variants are thought to mediate immune-driven hypersensitivity responses to asparaginase therapy [103,104]. Subsequent studies in long-term survivors of childhood ALL have further demonstrated that carriers of the *CEP72* risk allele have an increased risk of persistent motor neuropathy following vincristine exposure, supporting the role of this variant as a clinically relevant predictor of vincristine neurotoxicity [105]. Together, these findings highlight the growing potential of pharmacogenetic-guided approaches to improve toxicity risk stratification and personalize therapy in pediatric ALL.

In contrast, our variant-level meta-analyses of osteonecrosis yielded mixed results. The *MTHFR* rs1801133 polymorphism showed no significant association with osteonecrosis risk, while the *VDR* FokI (rs2228570) variant also demonstrated no consistent association and exhibited substantial between-study heterogeneity. Evidence for the role of *MTHFR* variants in osteonecrosis susceptibility, therefore, remains inconsistent [106,107]. A meta-analysis evaluating the MTHFR rs1801133 polymorphism across multiple populations similarly found no overall association with osteonecrosis of the femoral head, supporting the limited, context-dependent contribution of this variant to osteonecrosis risk [106]. In contrast, the *TYMS* enhancer repeat polymorphism showed a significant association with osteonecrosis risk in the pooled analysis of the present study; however, this finding was based on only two studies and should therefore be interpreted cautiously. Previous candidate-gene and genome-wide studies have suggested associations between osteonecrosis and variants in genes such as *SERPINE1*, *ACP1*, *BCL2L11*, and glutamate receptor pathways [32,34,36,48,55,56]. However, when considered collectively, the available evidence suggests that the contribution of individual genetic variants to osteonecrosis susceptibility is modest and likely modified by clinical factors. Indeed, clinical determinants such as age greater than 10 years, cumulative glucocorticoid exposure, and treatment protocol have consistently emerged as stronger predictors of osteonecrosis risk than individual genetic variants [36,49,56]. Genome-wide association studies have similarly struggled to identify variants that reach genome-wide significance thresholds for osteonecrosis susceptibility, underscoring the complex and multifactorial nature of this toxicity [107].

However, the substantial heterogeneity observed in the *NUDT15* rs116855232 meta-analysis (I^2^ = 86.5%) warrants careful consideration. The two included studies [30,66] differed in several respects: Choi et al. used a 6-mercaptopurine dose of 75 mg/m^2^/day in Korean patients, while Cao et al. used 60 mg/m^2^/day in Chinese patients. Background *TPMT* status was not uniformly reported, and ethnic differences may influence allele frequencies and other genetic modifiers. Despite this heterogeneity, pooling was considered appropriate for two reasons. First, both studies demonstrated a strong positive effect (individual ORs 5.25 and 65.07, both with CIs excluding 1), indicating that the heterogeneity is in magnitude rather than direction. Second, the random-effects model accounts for between-study variance and provides a conservative summary estimate. Sensitivity analysis confirmed that excluding either study did not alter the direction or significance of the pooled estimate.

Similarly, our meta-analysis evaluating the *MTHFR* c.677C>T polymorphism did not demonstrate a statistically significant association with methotrexate-induced myelosuppression. Moderate heterogeneity was observed across studies, likely reflecting differences in patient populations, treatment protocols, and definitions of toxicity. These findings are consistent with previous systematic reviews and meta-analyses that reported inconsistent, population-dependent associations between *MTHFR* variants and methotrexate toxicity [108]. Although the biological rationale linking folate pathway polymorphisms to methotrexate toxicity is well established, the variability in reported associations suggests that methotrexate-related toxicities are influenced by a complex interplay of genetic, environmental, and treatment-related factors. Consequently, current evidence does not support the routine clinical implementation of MTHFR genotyping to predict methotrexate toxicity.

Evidence for pharmacogenetic predictors of hepatotoxicity in pediatric ALL is also emerging. A recent genome-wide association study conducted within the AIEOP-BFM ALL cohort identified a strong association between variants within the *UGT1A* gene cluster and chemotherapy-related hyperbilirubinemia [109]. The rs6744284 variant demonstrated genome-wide significance and was strongly linked to the UGT1A1*28 and *37 alleles, which impair bilirubin glucuronidation and predispose patients to elevated bilirubin levels during chemotherapy [109]. These findings highlight the role of host hepatic metabolic pathways in treatment-related toxicity and suggest that genes involved in bilirubin metabolism may represent additional pharmacogenetic predictors of hepatotoxicity in pediatric ALL.

Recent meta-analytic evidence has also suggested that variants in methotrexate transport genes, particularly the reduced folate carrier *SLC19A1* (*RFC1*) G80A polymorphism, may contribute to increased risks of methotrexate-related toxicities in pediatric ALL, including myelosuppression and hepatotoxicity [110]. Furthermore, a recent meta-analysis by Yan et al. (2025) specifically examined the *ABCB1 C3435T* polymorphism and found a significant association with MTX-induced hepatotoxicity (OR 2.15, 95% CI 1.40–3.32), although no significant associations were observed for mucositis, myelosuppression, nephrotoxicity, or gastrointestinal toxicity [111]. Emerging evidence also suggests potential pharmacogenetic predictors of additional chemotherapy-related toxicities, such as oral mucositis. A recent candidate-gene analysis in pediatric patients receiving chemotherapy identified variants in transporter and metabolic genes, including *ABCC2*, *ABCC4*, *ABCC1*, and *MTHFR*, associated with an increased risk of chemotherapy-induced oral mucositis, although these findings require validation in larger cohorts [112]. Furthermore, emerging pathway-based pharmacogenomic analyses suggest that polygenic models incorporating multiple inflammatory and mucosal pathway genes may improve the prediction of methotrexate-induced mucositis in pediatric ALL [113]. Supporting this, a recent single-centre study by Larkin et al. developed polygenic toxicity risk scores using SNP-SNP combinations and found that three-SNP models improved the prediction of gastrointestinal, neurological, endocrine, and haematological toxicities compared with single-SNP approaches [114]. A summary of original research studies published after our search cutoff that are cited in the Discussion is provided in Supplementary Table S4. Beyond individual variants, combining genetic information into haplotypes or polygenic risk scores (PRS) may better capture the complex architecture of treatment-related toxicities. Single-variant approaches often miss additive or interactive effects, which may explain the inconsistent results in our meta-analyses of *MTHFR* and *VDR*. Emerging studies have shown that three-SNP PRS can improve the prediction of multiple toxicities compared with single-SNP models [114], and pathway-informed polygenic models enhance the prediction of methotrexate-induced mucositis [113]. Future research should prioritize the development and prospective validation of PRS that integrate clinical and genetic factors, alongside haplotype-based analyses, to move beyond the limitations of single-variant pharmacogenetics.

A consistent observation across the evaluated toxicities was substantial heterogeneity between studies. This heterogeneity likely reflects differences in treatment protocols, including variations among BFM, DFCI, COG, and NOPHO regimens; inconsistencies in toxicity definitions and grading systems; ethnic differences in allele frequencies; and variability in adjustment for clinical confounders. For example, associations between *MTHFR* polymorphisms and methotrexate toxicity varied considerably across populations, suggesting that contextual factors such as nutritional status, co-medications, and underlying genetic background may influence toxicity risk [39,53,68,74,75,79,84,88]. Such variability highlights the importance of harmonized study designs, standardized toxicity definitions, and adequately powered multi-ethnic studies in future pharmacogenetic research.

The application of the adapted STROPS checklist provided a structured framework for evaluating study quality. High-quality studies consistently reported validated genotyping methods, genotype frequencies, predefined genetic models, and toxicity outcomes defined according to NCI-CTCAE criteria [32,34,35,47,48,56]. In contrast, moderate-quality studies frequently lacked adequate reporting of Hardy–Weinberg equilibrium testing, multivariable adjustment for confounding factors, and standardized toxicity definitions [50,64,73,80,82]. These methodological limitations highlight the need for improved reporting standards and rigorous study design in pharmacogenetic investigations.

Population allele frequency analysis demonstrated strong concordance between the IndiGenomes dataset and global population reference datasets from gnomAD [25,26]. The observed correlations across populations, including South Asian and non-Finnish European groups, indicate that the pharmacogenetic variants identified in this systematic review are broadly consistent across diverse populations. Only a small proportion of variants showed statistically significant differences in allele frequency, suggesting that most pharmacogenetic markers associated with treatment-related toxicities in pediatric ALL are likely to have comparable population distributions. These findings support the potential generalisability of pharmacogenetic risk markers across populations, although population-specific studies remain important for validating clinical implementation. However, some pharmacogenetic markers exhibit pronounced population differences; for instance, the CYP2C19*2 allele (rs4244285) varies from 15% in Europeans to 30% in East Asians, underscoring that generalisability cannot be assumed for every variant. Therefore, while the variants prioritized in this review appear broadly concordant across the populations studied, careful validation in each target population remains essential, and the absence of major frequency differences for these particular variants should not be extrapolated to all pharmacogenetic markers. Additional comparison using UK Biobank data further supported the consistency of allele frequency distributions across populations [29].

Despite the strong allele frequency concordance observed between the IndiGenomes dataset and gnomAD South Asian populations (r = 0.997), it is important to emphasise that allele frequency alone does not validate clinical associations. South Asian populations were severely under-represented in the included studies, accounting for fewer than 8% of the 68 studies. Among the prioritised variants, only *NUDT15* rs116855232 and *TPMT* polymorphisms have been evaluated in small South Asian paediatric ALL cohorts (e.g., Indian and Bangladeshi studies) [53,82]. In contrast, the *TYMS* enhancer repeat (associated with osteonecrosis), *CEP72* (vincristine neuropathy), and *HLA* haplotypes (asparaginase hypersensitivity) have not been phenotype-validated in South Asian populations. This represents the largest evidence gap. The ongoing MPGx-INDALL study [11] is specifically designed to address this gap by prospectively evaluating these and other candidate variants in the Indian paediatric ALL cohort.

Overall, our findings suggest that pharmacogenetic evidence in pediatric ALL toxicity is uneven across drug classes. Robust and clinically actionable associations currently exist for thiopurine pharmacogenetics (*TPMT* and *NUDT15*), while emerging evidence supports pharmacogenetic predictors of vincristine neuropathy (*CEP72*) and asparaginase hypersensitivity (*HLA* variants). In contrast, genetic predictors of osteonecrosis and methotrexate-related toxicities remain inconsistent and require further validation. We finally identified a list of genes and genetic variants (see Supplementary Material S5) that will be evaluated in future observational genetic association studies [11]; some variants may be studied in specific ethnicities.

This study has several limitations. Although a comprehensive search strategy was employed, the possibility of publication bias and the exclusion of non-English studies cannot be completely ruled out. Quantitative meta-analysis was feasible for only a limited number of variants because many studies reported heterogeneous genetic models or lacked extractable genotype-level data. Furthermore, the majority of included studies were conducted in North America, Europe, and East Asia, with very few from Africa, South America, or South Asia, which may limit the generalisability of findings to under-represented populations. In addition, the included studies span several decades during which treatment protocols have evolved substantially, and residual confounding by clinical factors could not be fully addressed. Furthermore, many studies relied on candidate-gene approaches rather than genome-wide analyses, potentially limiting the discovery of novel genetic predictors. It should be noted, however, that most of these studies were underpowered for GWAS; conducting GWAS in small cohorts would not overcome this limitation but would instead generate many false-positive associations. Consequently, the reliance on candidate genes is as much a reflection of limited sample sizes as a methodological choice.

The selection criteria used in our study also differ from those used in CPIC guideline development [100]; therefore, some studies included in CPIC evidence synthesis were not captured in our systematic review. For example, CPIC includes studies in adult populations and mixed-age cohorts, accepts a broader range of toxicity definitions (not limited to NCI-CTCAE grading), and does not require the use of a formal quality assessment tool such as STROPS. As a result, some studies that informed the CPIC thiopurine dosing recommendations were not captured in our systematic review. In addition, our study represents one of the first systematic reviews in this field to apply the STROPS criteria for assessing the quality of pharmacogenetic studies, and our findings should be interpreted within this methodological context [15].

Despite these limitations, our findings have clear clinical implications. Strong evidence supports routine *TPMT* and *NUDT15* genotyping prior to thiopurine therapy, consistent with current pharmacogenetic guidelines [6]. Implementation studies have demonstrated the feasibility and clinical benefit of integrating genotype-guided thiopurine dosing into routine pediatric ALL treatment protocols, with evidence supporting improved toxicity management and favourable cost-effectiveness [115,116]. For variants such as *CEP72* and *HLA*, prospective studies are needed to determine whether genotype-guided therapy can improve treatment tolerability and clinical outcomes. In contrast, genetic testing for osteonecrosis and methotrexate-related toxicity should currently remain within the research domain. More broadly, accumulating real-world evidence suggests that pre-emptive pharmacogenomic testing can substantially reduce adverse drug reactions and improve medication safety across multiple therapeutic areas, supporting the integration of pharmacogenomics into routine clinical practice [117].

In this systematic review, we used automated data verification tools (Deben Services Ltd.) to assist with cross-checking extracted data. Recent evaluations have shown that while AI-assisted extraction can be effective for straightforward variables, its accuracy varies considerably across data domains and is limited by heterogeneity in reporting metrics [118,119]. The substantial heterogeneity we observed across studies—including variations in toxicity definitions, genetic models, reporting of allele frequencies, effect measures, and statistical presentation—substantially limited the feasibility of full automation, even with AI-assisted tools [120,121,122,123]. This experience underscores an important gap in the field: the lack of a harmonized, standardized reporting framework for pharmacogenetic association studies. Without uniform data presentation, efficient large-scale data extraction and synthesis, essential for meta-analyses and evidence-based guideline development, remain challenging [124,125]. The STROPS guideline provides a foundation, but its adoption remains limited, and the field would benefit from consensus on core data elements (e.g., genotype counts by genetic model, allele frequencies, Hardy–Weinberg equilibrium, adjusted effect estimates with confidence intervals, and standardised toxicity grading using NCI-CTCAE) to improve reproducibility and transparency and enable the creation of AI-driven pipelines for automated literature mining and meta-analysis [15,126]. Such standardization is urgently needed to accelerate evidence synthesis and translation in pharmacogenomics. Accordingly, a key message of our review is a call to the pharmacogenetics community to adopt harmonized reporting practices, thereby unlocking the full potential of artificial intelligence in evidence-based medicine [15,127].

Future research should prioritize harmonized toxicity definitions, protocol-specific analyses, multi-ethnic genome-wide meta-analyses, and the integration of germline genetic, somatic genomic, and clinical predictors. Based on the findings and limitations of this review, we propose a structured, phased roadmap to address existing controversies and knowledge gaps in paediatric ALL pharmacogenomics.

First, immediate priorities should focus on harmonised reporting. All future pharmacogenetic association studies should adhere to STROPS-based reporting standards, use NCI-CTCAE toxicity grading, clearly specify genetic models (dominant, recessive, or additive), and report genotype counts and Hardy–Weinberg equilibrium testing. This will enable future meta-analyses and reduce publication bias.

Second, short-term priorities should include replication of the strongest signals—particularly the *TYMS* enhancer repeat–osteonecrosis association, *CEP72*–vincristine neuropathy, and *HLA*–asparaginase hypersensitivity—in prospective, multi-ethnic cohorts, with a specific focus on South Asian and African populations, which are currently under-represented (<8% of studies).

Third, medium-term efforts should establish international consortia for adequately powered, multi-ethnic genome-wide association studies (GWAS), moving beyond candidate-gene approaches to enable the discovery of novel variants, while ensuring sufficient sample sizes to avoid false-positive findings.

Fourth, long-term goals should include prospective validation of polygenic risk scores (PRS) that integrate clinical and genetic predictors, followed by implementation in clinical decision support systems. International collaborative initiatives, such as the Ponte di Legno Toxicity Working Group [128], provide an important framework for generating adequately powered and standardised datasets that will be essential for advancing pharmacogenetic implementation in pediatric oncology.

## 5. Conclusions

This systematic review and meta-analysis provide a comprehensive evaluation of germline pharmacogenetic predictors of treatment-related toxicities in pediatric and adolescent acute lymphoblastic leukemia (ALL). Quantitative synthesis confirmed a strong association between the *NUDT15* rs116855232 variant and thiopurine-induced myelosuppression. It also identified a significant association between the *TYMS* enhancer repeat polymorphism and osteonecrosis risk, although this finding was based on only two studies and requires replication. In contrast, meta-analyses of *VDR* variants and *MTHFR* polymorphisms did not demonstrate significant associations with osteonecrosis or methotrexate-related myelosuppression, respectively. Evidence from multiple cohort studies further supports pharmacogenetic predictors of vincristine-induced neuropathy (*CEP72* rs924607) and asparaginase hypersensitivity (*HLA* haplotypes). Overall, substantial heterogeneity across studies underscores the urgent need for harmonised toxicity definitions, protocol-specific analyses, and multi-ethnic cohorts in pharmacogenetic research. Continued international collaboration and prospective pharmacogenomic studies will be essential to translate genetic risk markers into clinically actionable strategies that improve treatment safety, quality of life, and outcomes for children and adolescents with ALL.

## Figures and Tables

**Table 1:** Main characteristics of studies included in the systematic review (n = 42).

S.No	First Author (Year)	Country	Study Design	Type of ALL	Treatment Regimen	Genetic Analysis Strategy	Toxicity Investigated	Genes/Variants Investigated	Main Findings
**1**	Choi (2019) [30]	Republic of Korea	Retrospective cohort	B-ALL, T-ALL	OTH	C	Myelosuppression	*NUDT15*, *ABCC4*, *SLC29A1*, *SLC28A3*, *MOCOS*, *MTRR*, *SLCO1B1*, *IMPDH1*, *ADK*, *XDH*, *TYMP*	*NUDT15* rs116855232 was strongly associated with thiopurine-induced leukopenia and neutropenia; MOCOS and MTRR variants were linked to hepatotoxicity
**2**	Ansari (2012) [31]	Canada	Retrospective cohort	B-ALL, T-ALL	DFCI	C	Thrombocytopenia	*MRP2*, *MRP3*, *MRP5*	MRP transporter gene polymorphisms were associated with methotrexate-related thrombocytopenia and hepatic toxicity.
**3**	French (2008) [32]	USA	Retrospective cohort	B-ALL, T-ALL	CCG	C	Osteonecrosis	*PAI-1*, *TYMS*, *VDR*, *BGLAP*, *ESR1*, *LRP5*, *MTHFR*, *ABCB1*, *PTH*, *PTHR*, *ACP5*	PAI-1, VDR, and other bone metabolism–related gene variants were associated with osteonecrosis risk.
**4**	Ramsey (2017) [33]	USA	Prospective cohort	NA	SJCRH	G	Thrombosis	*F2RL1*	The *F2RL1* polymorphism was associated with increased steroid-related thrombosis risk.
**5**	Gagné (2019) [34]	Canada	Observational study	B-ALL, T-ALL	DFCI	G	Osteonecrosis	*ACP1*, *SH3YL1*, *CCND2*, *EBF1*, *GRID2*, *GRIK*, *GRIN3A*, *KCNMA1*, *KLF12*, *NARF*, *PCSK5*, *PROX1-AS1*, *SOX14*	Whole-exome sequencing and GWAS follow-up confirmed the implication of the ACP1-SH3YL1 locus in corticosteroid-induced osteonecrosis. Variants defining the *ACP1* B haplotype (e.g., rs79716074) were significantly associated with osteonecrosis risk, identifying a potentially causal variant for this complication.
**6**	Kishi (2007) [35]	USA	Observational study	NA	SJCRH	C	Infection and hyperbilirubinemia	*CYP3A5*, *RFC*, *VDR*, *GSTM1*, *UGT1A1*, *VDR*, *UGT1A1*, *CYP3A4* *GSTT1*, *MTHRF*, *RFC 80* *TPMT* (*238GG*; *460GG*; *719AA*; *others*), *TYMS enhancer repeat*, *UGT1A1 promoter repeat*, CYP3A5*3, *GSTM1*, *VDR intron8*	*CYP3A5*, *UGT1A1*, and VDR variants influenced infection risk and the development of hyperbilirubinemia during therapy.
**7**	Aplenc (2003) [36]	USA	Case–control	Mixed	CCG	C	Hepatotoxicity	*CYP3A*	*CYP3A* polymorphisms were evaluated for chemotherapy-related hepatotoxicity, with variable associations.
**8**	Yanagimachi (2013) [37]	Japan	Retrospective cohort	B-ALL, T-ALL	OTH	C	Nephrotoxicity	*SLC19A1*, *ABCG2*, *MTHFR*, *ABCC2*	*SLC19A1*, *ABCG2*, and *MTHFR* polymorphisms were investigated for nephrotoxicity, with no significant associations observed.
**9**	Razali (2020) [38]	Malaysia	Retrospective cohort	B-ALL, T-ALL	BFM	C	Myelosuppression	*ABCC2*, *ARID5B*	*ABCC2* and *ARID5B* variants were associated with increased risk of leukopenia.
**10**	Erčulj (2012) [39]	Slovenia	Retrospective cohort	Mixed	BFM	C	Myelosuppression	*MTHFR*, *MTHFD2*, *MTHFD1*, *TYMS*	*MTHFR*, *MTHFD1*, and *TYMS* polymorphisms were associated with methotrexate-related hematologic toxicity
**11**	Lund (2014) [40]	Denmark	Retrospective cohort	B-ALL, T-ALL	NOPHO	C	Infection	*VDR*, *TNF*, *IL10*, *IL4R*, *IL6*, *IL12RB1*	VDR and cytokine gene polymorphisms were associated with infection susceptibility during treatment.
**12**	Abaji (2018) [41]	Canada	Retrospective cohort	NA	NA	G	Neuropathy	*SYNE2*, *MRPL47*, *BAHD1*	Genome-wide variants (*SYNE2*, *MRPL47*, *BAHD1*) were associated with vincristine-induced peripheral neuropathy.
**13**	Gervasini (2017) [42]	Spain	Observational study	B-ALL, T-ALL	SHOP	C	Hematologic and hepatic toxicity	*SLC19A1 G80A*, *ABCB1 C1236T*, *ABCB1 G2677T/A*, *ABCB1 C3435T*, *ABCC2 C-24T*, *ABCC2 G1249A*, *ABCC2 IVS 23+56 T>C*, *ABCC4 T-1393C*, *ABCC4 C934A*, *ABCG2 C421A*	*SLC19A1*, *ABCB1*, *ABCC2*, *ABCC4*, and *ABCG2* variants were associated with methotrexate-related hematologic and hepatic toxicity.
	Gervasini (2017) [42]	Spain	Retrospective cohort	B-ALL, T-ALL	SHOP	C	Neutropenia	*DHFR*	DHFR polymorphism was associated with neutropenia during methotrexate therapy.
**14**	Wolthers (2017) [43]	Nordic	Case–control	B-ALL, T-ALL	NOPHO ALL	G	Pancreatitis	*ULK2*, *RGS6*, *GLTPD2*, *SPECC1*, *BBX*	Genome-wide variants, including ULK2 and RGS6, were associated with asparaginase-induced pancreatitis.
	Wolthers (2017) [43]	Nordic	Case–control	B-ALL, T-ALL	NOPHO	G	Osteonecrosis	*ULK2*, *MDFI*, *ABCA1*, *LINC01060*, *RGS6*	Genome-wide loci, including ULK2 and *ABCA1*, were associated with osteonecrosis susceptibility during ALL therapy.
**15**	Relling (2004) [44]	USA	Case–control	NA	SJCRH	C	Osteonecrosis	*CYP3A4*, *CYP3A5*, *MDR1*, *RFC*, *MTHFR*, *TYMS*, *VDR*, *UGT1A1*, *GSTM1*, *NR3C1*, *GSTP1*, *TPMT*, *GST*	*CYP3A*, *MTHFR*, VDR, and related variants were associated with osteonecrosis risk.
**16**	Kutszegi (2015) [45]	Hungary	Retrospective cohort	B-ALL, T-ALL	BFM	C	Delayed clearance	*GRIA1*, *GALNT10*	*GRIA1* variants were associated with asparaginase-related hypersensitivity reactions.
**17**	Cheng (2021) [46]	China	Case-control	B-ALL, T-ALL	CCLG	C	Delayed methotrexate clearance	*SLCO1B1*	*SLCO1B1* polymorphism was associated with delayed methotrexate clearance.
**18**	Kahn (2018) [47]	USA/Canada	Prospective cohort	B-ALL, T-ALL	DFCI	Exploratory population genetics	Osteonecrosis, bone fracture, overall toxicities, survival outcomes	*Not specified*	Ethnicity-based differences were observed in osteonecrosis and survival, with Hispanic patients demonstrating lower osteonecrosis risk but inferior survival; exploratory analyses suggested differences in variant frequencies between ethnic groups.
**19**	Burgueño-Rodríguez (2020) [48]	Uruguay	Retrospective cohort	NA	BFM	C	Myelosuppression	*TPMT*	*TPMT* variants were associated with hematologic toxicity during therapy.
**20**	Hareedy (2015) [49]	Egypt	Prospective cohort	NA	MSJT	C	Myelosuppression/Cytopenias	*ITPA*, *IMPDH1*, *SLC28A2*, *SLC28A3*, *SLC29A1*, *ABCC4*	*ITPA*, *IMPDH1*, and nucleoside transporter gene variants were associated with myelosuppression/cytopenias.
**21**	Ben Tanfous (2015) [50]	Canada	Retrospective cohort	NA	DFCI	C	Asparaginase-related hypersensitivity and pancreatitis	*ASNS*	*ASNS* triple-repeat (3R3R) genotype was significantly associated with increased risk of asparaginase-related allergies and pancreatitis, whereas haplotype *1 (2R) showed a protective effect.
**22**	Xue (2015) [51]	China	Retrospective cohort	B-ALL, T-ALL	OTH	C	Hepatotoxicity	*CCND1*	The *CCND1* variant was associated with methotrexate-related hepatotoxicity.
**23**	Li L(2019) [52]	USA	prospective cohort	NA	NA	G	Neuropathy	*COCH*, *intergenic loci*	Genome-wide variants (including *COCH* and intergenic loci) were associated with vincristine-induced neuropathy.
**24**	Zaman (2019) [53]	Bangladesh	prospective cohort	NA	UKALL	C	Hypersensitivity	*ITPA*	ITPA polymorphism was associated with increased myelosuppression risk.
**25**	Højfeldt (2019) [54]	Nordic	Retrospective cohort	B-ALL, T-ALL	NOPHO	G	Neuropathy	*HLA-DQA1*, *CNOT3*, *TAP2*	*HLA-DQA1* and related loci were associated with asparaginase hypersensitivity.
**26**	Diouf (2015) [55]	USA	Retrospective cohort	NA	SJCRH	G	Vincristine-induced peripheral neuropathy	*CEP72*, *ETAA1*, *MTNR1B*, *TMEM215*, *NDUFA6*	The *CEP72* variant was strongly associated with vincristine-induced neuropathy.
**27**	Kawedia (2011) [56]	USA	Prospective cohort	NA	SJCRH	G	Osteonecrosis	*SH3YL1*, *ACP1*, *SLC12A8*, *SLCO2A1*, *OR5M9*, *NUDT15*, *CRNN*, *CNTNAP2*	Genome-wide loci, including *SH3YL1* and *ACP1*, were associated with osteonecrosis and pharmacokinetic variability.
**28**	Karol (2016) [57]	USA	Prospective cohort	NA	NA	G	Osteonecrosis	*BMP7*, *PROX1-AS1*, *LINC00251*, *DOK5*, *GRID2*	*BMP7* and related loci were associated with early-onset osteonecrosis.
**29**	Moulik N R(2015) [58]	India	Prospective cohort	B-ALL, T-ALL	CCG	C	Steroid toxicity	*MTHFR*	*MTHFR* polymorphism was associated with induction-phase myelosuppression.
**30**	Kaymak-Cihan (2017) [59]	Turkey	Retrospective cohort	NA	BFM	C	Pancreatitis	*NR3C1*	*NR3C1* polymorphism was associated with steroid-related adverse effects.
**31**	Abaji (2017) [60]	Canada/USA	Retrospective cohort	NA	DFCI	EWAS	Myelosuppression	*SLC7A13*, *MYBBP1A*, *YTHDC2*, *ADAMTS17*, *MYBBP1A*, *SPECC1*, *PKD2L1*, *RIN3*, *SPEF2*, *SLC39A12*, *MPEG1*, *IL16*	Genome-wide variants (including *SLC7A13*) were associated with pancreatitis risk.
**32**	Liu SG (2011) [61]	China	Retrospective cohort	B-ALL, T-ALL	CCLG	C	Myelosuppression hepatotoxicity	*MTHFR*	*MTHFR* polymorphism was associated with consolidation-phase myelosuppression
**33**	Tantawy AA (2010) [62]	Egypt	Prospective cohort	B-ALL, T-ALL	MTX-based	C	Hepatic toxicity, mucositis, neutropenia (MTX-related)	*MTHFR* (*c.677C>T*, *1298c.A>C*)	*MTHFR* 677TT genotype was significantly associated with grade III/IV methotrexate-related toxicity
**34**	Plesa (2019) [63]	Canada	Retrospective cohort	NA	DFCI	C	Osteonecrosis	*BCL2L11*	The *BCL2L11* variant was associated with susceptibility to osteonecrosis.
**35**	Karol (2015) [64]	NA	Retrospective cohort	NA	NA	G	Osteonecrosis	*GRIN3A*, *GRIK1*	*GRIN3A* and *GRIK1* loci were associated with glucocorticoid-related osteonecrosis.
**36**	Sepe (2012) [65]	USA	Nested Case -control	NA	BFM	C	Myelosuppression	*CYP3A4*, *CYP3A5*, *GSTP1*, *GST*, *UGT1A1*, *DHFR*, *MS*, *MTHFR* *MTRR*, *RFC1*, *SHMT*, *TPMT*, *TS*	*CYP3A4*, *GSTP1*, and folate pathway gene variants were associated with infection and toxicity outcomes.
**37**	Cao (2020) [66]	China	Prospective cohort	NA	CCCG	E	Myelosuppression	*NUDT15*, *TPMT*, *CYP2A7*, *COMT*	*NUDT15* and *TPMT* variants were associated with mercaptopurine-induced myelosuppression.
**38**	Adam de Beaumais T et al. (2011) [67]	France	Retrospective cohort		European Organisation for Research and Treatment of Cancer (EORTC) 58951 protocol	C	Survival and mucositis	*TPMT*, *ITPA*	*TPMT* and *ITPA* polymorphisms were associated with methotrexate-related hepatotoxicity.
**39**	Liu (2013) [68]	NA	Retrospective cohort	NA	NA	C	Hematologic toxicity	*FPGS*	*FPGS* polymorphisms were associated with methotrexate-related hematologic toxicity and clinical outcomes.
**40**	Gregers (2015) [69]	Denmark	Retrospective cohort	NA	NOPHO	C	Bone marrow toxicity during induction Hepatotoxicity after high-dose methotrexate Relapse risk (survival outcome)	*ABCA1*	*ABCB1* 1199G>A variant was associated with increased relapse risk, while the *ABCB1* 3435TT genotype was associated with increased bone marrow toxicity during induction therapy and the 3435CC genotype with increased methotrexate-related hepatotoxicity.
**41**	Karas-Kuželički (2016) [70]	Slovenia	Retrospective cohort	NA	BFM	C	Symptomatic osteonecrosis	*TPMT*, *MTHFR*, *ESR1*, *COL1A1*	Older age and more recent treatment protocols were independent risk factors for osteonecrosis. In addition, *TPMT* polymorphisms modulated osteonecrosis risk in older children, while ESR1 variants were associated with osteonecrosis in younger patients.
**42**	Kutszegi (2021) [71]	Hungary	Retrospective cohort	NA	NA	C		*HLA-DRB1*	*HLA-DRB1* polymorphisms were associated with asparaginase hypersensitivity.

BFM: Berlin–Frankfurt–Münster; DFCI: Dana–Farber Cancer Institute; SJCRH: St. Jude Children’s Research Hospital; NOPHO: Nordic Society of Paediatric Haematology and Oncology; CCG: Children’s Cancer Group; CCLG: Chinese Children’s Leukemia Group; SHOP: Spanish Society of Pediatric Hematology and Oncology; UKALL: United Kingdom Acute Lymphoblastic Leukemia; EORTC: European Organisation for Research and Treatment of Cancer; OTH: Other protocol; G: genome-wide; C: candidate gene; EWAS, exome-wide association study.

**Table 2:** Genetic associations with treatment-related adverse effects in pediatric acute lymphoblastic leukaemia.

Study	Adverse Effect	Gene	SNP/Variant	Genetic Model	Risk Genotype	Comparator	OR (95% CI)
**Choi R et al. 2019** [30]	Leukopenia	*NUDT15*	rs116855232	Dominant	CT+TT	CC	10.7 (1.96–58.37)
**Choi R et al. 2019** [30]	Neutropenia	*NUDT15*	rs116855232	Dominant	CT+TT	CC	2.91 (0.62–13.82)
**Choi R et al. 2019** [30]	Leukopenia	*ABCC4*	rs2274405	Dominant	CT+TT	CC	1.12 (0.21–6.04)
**Choi R et al. 2019** [30]	Leukopenia	*ABCC4*	rs2274406	Dominant	CT+TT	CC	0.78 (0.16–3.63)
**Choi R et al. 2019** [30]	Neutropenia	*ABCC4*	rs4773866	Dominant	CT+TT	CC	3.66 (0.64–20.79)
**Choi R et al. 2019** [30]	Hepatotoxicity	*MOCOS*	rs3744900	Dominant	GA+AA	GG	3.46 (1.33–9.00)
**Choi R et al. 2019** [30]	Hepatotoxicity	*MTRR*	rs1801394	Dominant	AG+GG	AA	0.33 (0.16–0.67)
**Kishi S et al. 2007** [35]	Infection	*CYP3A5*	*3	Dominant	GA+AA	GG	0.23 (0.08–0.69)
**Kishi S et al. 2007** [35]	Infection	*RFC*	80A>G	Dominant	AG+GG	AA	3.2 (0.12–8.5)
**Kishi S et al. 2007** [35]	Infection	*VDR*	intron 8 G>A	Dominant	GA+AA	GG	0.4 (0.2–0.9)
**Kishi S et al. 2007** [35]	Hyperbilirubinemia	*UGT1A1*	promoter repeat	Dominant	Variant	WT	14.9 (7.3–30.5)
**Kishi S et al. 2007** [35]	Hyperbilirubinemia	*CYP3A5*	*3	Dominant	GA+AA	GG	0.3 (0.1–0.6)
**Kishi S et al. 2007** [35]	Hyperbilirubinemia	*CYP3A4*	*1B	Dominant	AG+GG	AA	3.7 (1.4–9.9)
**Yanagimachi M et al. 2013** [37]	Nephrotoxicity	*SLC19A1*	rs1051266	Dominant	AG+GG	AA	1.0 (0.5–2.1)
**Yanagimachi M et al. 2013** [37]	Nephrotoxicity	*ABCG2*	rs2231142	Dominant	CA+AA	CC	0.7 (0.3–1.4)
**Yanagimachi M et al. 2013** [37]	Nephrotoxicity	*MTHFR*	rs1801133	Dominant	CT+TT	CC	0.6 (0.3–1.2)

## Data Availability

No new data were created or analyzed in this study.

## Supplementary Materials

The following supporting information can be downloaded at: https://www.mdpi.com/article/10.3390/ph19081204/s1, Supplementary Material S1: Search strategies; Supplementary Material S2: Link for CADIMA tool; Supplementary Material S3: Data extraction template; Supplementary Material S4: Quality assessment—STROPS checklist; Supplementary Material S5: List of genes and genetic variants (with rs IDs); Supplementary Table S1. Detailed characteristics of included studies (n = 68); Supplementary Table S2. Quality assessment—STROPS scores; Supplementary Table S3. Pearson correlation analysis of minor allele frequencies; Supplementary Table S4. Original research studies published after the primary search cutoff; Supplementary Table S5. Mapping of STROPS checklist items; Supplementary Figure S1. Distribution of STROPS quality assessment scores; Supplementary Figure S2. Fisher’s exact test—allele frequency comparison; Supplementary Figure S3. UK Biobank allele frequency comparison.
