# Supplementary material for: Pharmacogenetic Predictors of Chemotherapy Treatment-Related Toxicities in Paediatric and Adolescent Acute Lymphoblastic Leukemia: A Systematic Review, Meta-Analysis and Literature-Based Candidate Prioritization
Source: Pharmaceuticals (Basel). 2026 Aug 1;19(8):1204. doi: 10.3390/ph19081204 (PMC13516592; doi:10.3390/ph19081204)
Supplement: Supplementary file 1 [file pharmaceuticals-19-01204-s001.zip › pharmaceuticals-4354157-supplementary.pdf]

Table of Contents

Supplementary Material S1: Search strategies

Supplementary Material S2: Link for CADIMA software

Supplementary Material S3: Data extraction template

Supplementary Material S4: Quality assessment – STROPS checklist

Supplementary Material S5: List of genes and genetic variants (with rs IDs)

Supplementary Table S1. Detailed characteristics of included studies (n = 68)

Supplementary Table S2. Quality assessment – STROPS scores

Supplementary Table S3. Pearson correlation analysis of minor allele frequencies

Supplementary Table S4. Original research studies published after the primary search cutoff

Supplementary Table S5 – Mapping of STROPS checklist items

Supplementary Figure S1. Distribution of STROPS quality assessment scores

Supplementary Figure S2. Fisher's exact test – allele frequency comparison

Supplementary Figure S3. UK Biobank allele frequency comparison

Supplementary Material S1: Search strategies

PUBMED:

(Acute lymphoblastic leukemia[Title/Abstract] OR Precursor Cell Lymphoblastic Leukemia-Lymphoma[MeSH Terms] OR acute lymphocytic leukemia[Title/Abstract] OR acute leukemia[Title/Abstract] OR acute lymphoid leukemia[Title/Abstract] OR childhood leukemia[Title/Abstract])

AND

(Vincristine[Title/Abstract] OR Anthracycline*[Title/Abstract] OR Daunorubicin[Title/Abstract] OR Doxorubicin[Title/Abstract] OR Mitoxantrone[Title/Abstract] OR 6-Mercaptopurine[Title/Abstract] OR Methotrexate[Title/Abstract] OR Asparaginase[Title/Abstract] OR Glucocorticoid*[Title/Abstract] OR Dexamethasone[Title/Abstract] OR Prednisolone[Title/Abstract] OR Steroid*[Title/Abstract] OR Cyclophosphamide[Title/Abstract] OR Cytarabine[Title/Abstract] OR Induction Chemotherapy[Title/Abstract] OR Maintenance Chemotherapy[Title/Abstract] OR Consolidation Chemotherapy[Title/Abstract] OR Vincristine[MeSH Terms] OR Anthracyclines[MeSH Terms] OR Daunorubicin[MeSH Terms] OR Doxorubicin[MeSH Terms] OR Mitoxantrone[MeSH Terms] OR Mercaptopurine[MeSH Terms] OR Methotrexate[MeSH Terms] OR Asparaginase[MeSH Terms] OR Glucocorticoids[MeSH Terms] OR Dexamethasone[MeSH Terms] OR Prednisolone[MeSH Terms] OR Steroids[MeSH Terms] OR Cyclophosphamide[MeSH Terms] OR Cytarabine[MeSH Terms] OR Induction Chemotherapy[MeSH Terms] OR Maintenance Chemotherapy[MeSH Terms] OR Consolidation Chemotherapy[MeSH Terms] OR Berlin-Frankfurt-Münster[Title/Abstract] OR BFM[Title/Abstract] OR Children's Oncology Group[Title/Abstract] OR COG[Title/Abstract] OR AALL[Title/Abstract] OR GRAALL[Title/Abstract] OR CALGB[Title/Abstract] OR Dutch Childhood Oncology Group[Title/Abstract] OR DCOG[Title/Abstract] OR Dana Farber Cancer Institute Consortium[Title/Abstract] OR DFCI[Title/Abstract] OR United Kingdom Acute Lymphoblastic Leukaemia[Title/Abstract] OR UKALL[Title/Abstract] OR MRC UK[Title/Abstract] OR St Jude[Title/Abstract] OR TOTXV[Title/Abstract] OR Nordic Society[Title/Abstract] OR NOPHO[Title/Abstract] OR thiopurine*[Title/Abstract] OR Antifolate*[Title/Abstract] OR Vinca Alkaloid*[Title/Abstract] OR Alkylating agent*[Title/Abstract] OR Alkylator*[Title/Abstract] OR Pyrimidine*[Title/Abstract] OR Antineoplastic Agents, Alkylating[MeSH Terms] OR Antimetabolites, Antineoplastic[MeSH Terms] OR Antibiotics, Antineoplastic[MeSH Terms])

AND

(Genetic polymorphism*[Title/Abstract] OR Single nucleotide polymorphism*[Title/Abstract] OR Genetic variant*[Title/Abstract] OR Pharmacogen*[Title/Abstract] OR Mutation*[Title/Abstract] OR Personalized Medicine[Title/Abstract] OR Polymorphism*[Title/Abstract] OR Variant*[Title/Abstract] OR Precision medicine[Title/Abstract] OR germline mutation[Title/Abstract] OR genetic association study[Title/Abstract] OR genotype[Title/Abstract] OR genetic background[Title/Abstract] OR genotyping technique[Title/Abstract] OR genetic heterogeneity[Title/Abstract] OR genetic profile[Title/Abstract] OR Polymorphism, Genetic[MeSH Terms] OR Pharmacogenomic Variants[MeSH Terms] OR Polymorphism,

Single Nucleotide[MeSH Terms] OR Pharmacogenetics[MeSH Terms] OR Genome-Wide Association Study[MeSH Terms] OR Genomics/methods[MeSH Terms] OR Germ-Line Mutation[MeSH Terms] OR Mutation[MeSH Terms] OR Precision Medicine[MeSH Terms] OR Genetic Association Studies[MeSH Terms] OR Genotype[MeSH Terms] OR Alleles[MeSH Terms])

AND

(Drug-Related Side Effects and Adverse Reactions[MeSH Terms] OR Heart failure[MeSH Terms] OR Chemical and Drug Induced Liver Injury[MeSH Terms] OR Chemotherapy-Related Cognitive Impairment[MeSH Terms] OR Drug Hypersensitivity[MeSH Terms] OR Leukopenia[MeSH Terms] OR Agranulocytosis[MeSH Terms] OR Neutropenia[MeSH Terms] OR Febrile Neutropenia[MeSH Terms] OR Chemotherapy-Induced Febrile Neutropenia[MeSH Terms] OR Anemia[MeSH Terms] OR Thrombocytopenia[MeSH Terms] OR Polyneuropathy[MeSH Terms] OR Thromboembolism[MeSH Terms] OR Embolism, Paradoxical[MeSH Terms] OR Intracranial Embolism and Thrombosis[MeSH Terms] OR Carotid Artery Thrombosis[MeSH Terms] OR Intracranial Embolism[MeSH Terms] OR Intracranial Thrombosis[MeSH Terms] OR Venous Thromboembolism[MeSH Terms] OR Pancreatitis[MeSH Terms] OR Amylase[MeSH Terms] OR Lipase[MeSH Terms] OR Hyperglycemia[MeSH Terms] OR Hepatic Insufficiency[MeSH Terms] OR Liver Failure[MeSH Terms] OR End Stage Liver Disease[MeSH Terms] OR Hepatic Encephalopathy[MeSH Terms] OR Liver Failure, Acute[MeSH Terms] OR Acute kidney injury[MeSH Terms] OR Treatment outcome[MeSH Terms] OR Survival Rate[MeSH Terms] OR Overall survival[MeSH Terms] OR Disease-Free Survival[MeSH Terms] OR Adverse drug reaction*[Title/Abstract] OR Chemotherapy related toxicit*[Title/Abstract] OR Drug toxicit*[Title/Abstract] OR Adverse effect*[Title/Abstract] OR Toxicit*[Title/Abstract] OR Peripheral neuropath*[Title/Abstract] OR Neuropath*[Title/Abstract] OR Polyneuropathy[Title/Abstract] OR Myelosuppression[Title/Abstract] OR Hypersensitivit*[Title/Abstract] OR Allergic reaction*[Title/Abstract] OR Osteonecrosis[Title/Abstract] OR Hepatotoxicity[Title/Abstract] OR Nephrotoxicity[Title/Abstract] OR Heart failure[Title/Abstract] OR Drug Hypersensitivit*[Title/Abstract] OR Leukopenia[Title/Abstract] OR Agranulocytosis[Title/Abstract] OR Neutropenia[Title/Abstract] OR Febrile Neutropenia[Title/Abstract] OR Chemotherapy-Induced Febrile Neutropenia[Title/Abstract] OR Anemia[Title/Abstract] OR Thrombocytopenia[Title/Abstract] OR Thrombosis[Title/Abstract] OR Thromboembolism[Title/Abstract] OR Thromboembolic event*[Title/Abstract] OR Intracranial Embolism Thrombosis[Title/Abstract] OR Carotid Artery Thrombosis[Title/Abstract] OR Intracranial Embolism[Title/Abstract] OR Intracranial Thrombosis[Title/Abstract] OR Venous Thromboembolism[Title/Abstract] OR Pancreatitis[Title/Abstract] OR Acute pancreatitis[Title/Abstract] OR Amylase[Title/Abstract] OR Lipase[Title/Abstract] OR Hyperglycemia[Title/Abstract] OR Drug induced diabetes[Title/Abstract] OR Hepatic Insufficiency[Title/Abstract] OR Liver Failure[Title/Abstract] OR End Stage Liver Disease[Title/Abstract] OR Hepatic Encephalopathy[Title/Abstract] OR Acute liver failure[Title/Abstract] OR Acute kidney injury[Title/Abstract] OR Treatment outcome[Title/Abstract] OR Survival Rate[Title/Abstract] OR Overall survival[Title/Abstract] OR Disease-Free

Survival[Title/Abstract] OR Leukemia free survival[Title/Abstract] OR Relapse free survival[Title/Abstract])

NOT

(Bone Marrow Transplant*[Title/Abstract] OR Stem Cell Transplant*[Title/Abstract] OR Stem Cell Transplantation[MeSH Terms] OR Cell transplantation[MeSH Terms] OR Bone Marrow Transplantation[MeSH Terms] OR Hematopoietic Stem Cell Mobilization[MeSH Terms] OR Transplants[MeSH Terms] OR Allografts[MeSH Terms] OR Autografts[MeSH Terms] OR Leukemia, Lymphocytic, Chronic, B-Cell[MeSH Terms] OR Leukemia, Prolymphocytic, B-Cell[MeSH Terms] OR Leukemia, Prolymphocytic, B-Cell[MeSH Terms] OR Leukemia, Prolymphocytic, T-Cell[MeSH Terms] OR Leukemia-Lymphoma, Adult T-Cell[MeSH Terms] OR Leukemia, Large Granular Lymphocytic[MeSH Terms] OR Leukemia, Prolymphocytic, T-Cell[MeSH Terms] OR adult leukemia[Title/Abstract] OR chronic leukemia[Title/Abstract] OR leukemia, myeloid, acute[MeSH Terms] OR acute myeloid leukemia[Title/Abstract])

NOT

(Case Reports[Publication Type] OR Clinical Conference[Publication Type] OR Editorial[Publication Type] OR Interview[Publication Type] OR Meta-Analysis[Publication Type] OR Practice Guideline[Publication Type] OR Review[Publication Type] OR Systematic Review[Publication Type] OR Retracted Publication[Publication Type])

AND

("1995/01/10"[Date - Publication] : "2021/01/10"[Date - Publication])

Embase:

('acute lymphoblastic leukemia':ab,ti OR 'acute lymphocytic leukemia':ab,ti OR 'acute leukemia':ab,ti OR 'childhood leukemia':ab,ti OR 'acute lymphoblastic leukemia'/exp)

AND

('vincristine'/exp OR 'anthracycline'/exp OR 'daunorubicin'/exp OR 'doxorubicin'/exp OR 'mitoxantrone'/exp OR 'mercaptopurine'/exp OR 'methotrexate'/exp OR 'asparaginase'/exp OR 'glucocorticoid'/exp OR 'dexamethasone'/exp OR 'prednisolone'/exp OR 'steroid'/exp OR 'cyclophosphamide'/exp OR 'cytarabine'/exp OR 'induction chemotherapy'/exp OR 'maintenance chemotherapy'/exp OR 'consolidation chemotherapy'/exp OR 'anthracycline*':ab,ti OR daunorubicin:ab,ti OR doxorubicin:ab,ti OR mitoxantrone:ab,ti OR '6 mercaptopurine':ab,ti OR methotrexate:ab,ti OR asparaginase:ab,ti OR 'glucocorticoid*':ab,ti OR dexamethasone:ab,ti OR prednisolone:ab,ti OR 'steroid*':ab,ti OR cyclophosphamide:ab,ti OR cytarabine:ab,ti OR 'induction chemotherapy':ab,ti OR 'maintenance chemotherapy':ab,ti OR 'consolidation chemotherapy':ab,ti OR vincristine:ab,ti OR 'berlin frankfurt münster':ab,ti OR 'children oncology group':ab,ti OR cog:ab,ti OR aall:ab,ti OR graall:ab,ti OR calgb:ab,ti OR 'dutch childhood oncology group':ab,ti OR dcog:ab,ti OR 'dana farber cancer institute consortium':ab,ti OR dfci:ab,ti OR 'united kingdom acute lymphoblastic leukaemia':ab,ti OR ukall:ab,ti OR 'mrc uk':ab,ti OR 'st jude':ab,ti OR totxv:ab,ti OR 'nordic society':ab,ti OR nopho:ab,ti OR thiopurine*:ab,ti OR

antifolate*:ab,ti OR 'vinca alkaloid*':ab,ti OR 'alkylating agent*':ab,ti OR alkylator*:ab,ti OR pyrimidine*:ab,ti OR 'alkylating agent':ab,ti OR 'antineoplastic antimetabolite'/exp OR 'antineoplastic antibiotic'/exp)

AND

('genetic polymorphism'/exp OR 'single nucleotide polymorphism'/exp OR 'genetic variability'/exp OR 'pharmacogenetics'/exp OR 'mutation'/exp OR 'personalized medicine'/exp OR 'germline mutation'/exp OR 'genetic association study'/exp OR 'genotype'/exp OR 'genetic background'/exp OR 'genotyping technique'/exp OR 'genetic heterogeneity'/exp OR 'genetic profile'/exp OR 'allele'/exp OR 'genomics'/exp OR 'genetic polymorphism*':ab,ti OR 'single nucleotide polymorphism*':ab,ti OR 'genetic variant*':ab,ti OR pharmacogen*:ab,ti OR mutation*:ab,ti OR 'personalized medicine':ab,ti OR polymorphism*:ab,ti OR variant*:ab,ti OR 'precision medicine':ab,ti OR 'germline mutation':ab,ti OR 'genetic association study':ab,ti OR genotype:ab,ti OR 'genetic background':ab,ti OR 'genotyping technique':ab,ti OR 'genetic heterogeneity':ab,ti OR 'genetic profile':ab,ti)

AND

('adverse drug reaction'/exp OR 'heart failure'/exp OR 'toxic hepatitis'/exp OR 'chemotherapy-related cognitive impairment'/exp OR 'drug hypersensitivity'/exp OR 'leukopenia'/exp OR 'agranulocytosis'/exp OR 'neutropenia'/exp OR 'febrile neutropenia'/exp OR 'anemia'/exp OR 'thrombocytopenia'/exp OR 'polyneuropathy'/exp OR 'pancreatitis'/exp OR 'hyperglycemia'/exp OR 'liver failure'/exp OR 'end stage liver disease'/exp OR 'hepatic encephalopathy'/exp OR 'acute liver failure'/exp OR 'acute kidney failure'/exp OR 'treatment outcome'/exp OR 'survival rate'/exp OR 'overall survival'/exp OR 'disease free survival'/exp OR 'recurrence free survival'/exp OR 'cancer specific survival'/exp OR 'adverse drug reaction*':ab,ti OR 'chemotherapy related toxicit*':ab,ti OR 'drug toxicit*':ab,ti OR 'adverse effect':ab,ti OR toxicity:ab,ti OR 'peripheral neuropathy':ab,ti OR neuropathy:ab,ti OR polyneuropathy:ab,ti OR myelosuppression:ab,ti OR hypersensitivity:ab,ti OR 'allergic reaction':ab,ti OR osteonecrosis:ab,ti OR hepatotoxicity:ab,ti OR nephrotoxicity:ab,ti OR 'heart failure':ab,ti OR 'drug hypersensitivity*':ab,ti OR leukopenia:ab,ti OR agranulocytosis:ab,ti OR neutropenia:ab,ti OR 'febrile neutropenia':ab,ti OR 'chemotherapy-induced febrile neutropenia':ab,ti OR anemia:ab,ti OR thrombocytopenia:ab,ti OR thrombosis:ab,ti OR thromboembolism:ab,ti OR 'thromboembolic event':ab,ti OR 'intracranial embolism thrombosis':ab,ti OR 'carotid artery thrombosis':ab,ti OR 'intracranial embolism':ab,ti OR 'intracranial thrombosis':ab,ti OR 'venous thromboembolism':ab,ti OR pancreatitis:ab,ti OR 'acute pancreatitis':ab,ti OR amylase:ab,ti OR hyperglycemia:ab,ti OR 'drug induced diabetes':ab,ti OR 'hepatic insufficiency':ab,ti OR 'liver failure':ab,ti OR 'end stage liver disease':ab,ti OR 'hepatic encephalopathy':ab,ti OR 'acute liver failure':ab,ti OR 'acute kidney injury':ab,ti OR 'treatment outcome':ab,ti OR 'survival rate':ab,ti OR 'overall survival':ab,ti OR 'disease-free survival':ab,ti OR 'leukemia free survival':ab,ti OR 'relapse free survival':ab,ti OR 'paradoxical embolism'/exp OR 'brain embolism'/exp OR 'thromboembolism'/exp OR 'carotid artery thrombosis'/exp OR 'occlusive cerebrovascular disease'/exp OR 'venous thromboembolism'/exp OR 'amylase'/exp OR 'triacylglycerol lipase'/exp)

NOT

('bone marrow transplant*':ab,ti OR 'stem cell transplant*':ab,ti OR 'acute myeloid leukemia':ab,ti OR 'adult leukemia':ab,ti OR 'chronic leukemia':ab,ti OR 'stem cell transplantation'/exp OR 'cell transplantation'/exp OR 'bone marrow transplantation'/exp OR 'stem cell mobilization'/exp OR 'transplantation'/exp OR 'allograft'/exp OR 'autograft'/exp OR 'chronic b-cell leukemia cell line'/exp OR 't cell leukemia'/exp OR 'large granular lymphocyte leukemia'/exp OR 'prolymphocytic leukemia'/exp OR 'acute myeloid leukemia'/exp)

NOT

('case reports':it OR 'clinical conference':it OR editorial:it OR interview:it OR 'meta analysis':it OR practice:it OR guideline:it OR review:it OR 'systematic review':it OR 'retracted publication':it)

AND

[10-1-1995]/sd NOT [11-1-2021]/sd

Scopus:

(("Acute lymphoblastic leukemia") OR ("Acute lymphoblastic leukaemia") OR INDEXTERMS ("Precursor Cell Lymphoblastic Leukemia-Lymphoma") OR ("acute lymphocytic leukemia") OR ("acute leukemia") OR ("acute lymphoid leukemia") OR ("childhood leukaemia") OR ("childhood leukemia")) AND ("Vincristine") OR ("Anthracycline*") OR ("Daunorubicin") OR ("Doxorubicin") OR ("Mitoxantrone") OR ("6-Mercaptopurine") OR ("Methotrexate") OR ("Asparaginase") OR ("Glucocorticoid*") OR ("Dexamethasone") OR ("Prednisolone") OR ("Steroid*") OR ("Cyclophosphamide") OR ("Cytarabine") OR ("Induction Chemotherapy") OR ("Maintenance Chemotherapy") OR ("Consolidation Chemotherapy") OR INDEXTERMS ("Vincristine") OR INDEXTERMS ("Anthracyclines") OR INDEXTERMS ("Daunorubicin") OR INDEXTERMS ("Doxorubicin") OR INDEXTERMS ("Mitoxantrone") OR INDEXTERMS ("Mercaptopurine") OR INDEXTERMS ("Methotrexate") OR INDEXTERMS ("Asparaginase") OR INDEXTERMS ("Glucocorticoids") OR INDEXTERMS ("Dexamethasone") OR INDEXTERMS ("Prednisolone") OR INDEXTERMS ("Steroids") OR INDEXTERMS ("Cyclophosphamide") OR INDEXTERMS ("Cytarabine") OR INDEXTERMS ("Induction Chemotherapy") OR INDEXTERMS ("Maintenance Chemotherapy") OR INDEXTERMS ("Consolidation Chemotherapy") OR ("Berlin-Frankfurt-Münster") OR ("BFM") OR ("Children's Oncology Group") OR ("COG") OR ("AALL") OR ("GRAALL") OR ("CALGB") OR ("Dutch Childhood Oncology Group") OR ("DCOG") OR ("Dana Farber Cancer Institute Consortium") OR ("DFCI") OR ("United Kingdom Acute Lymphoblastic Leukaemia") OR ("UKALL") OR ("MRC UK") OR ("St Jude") OR ("TOTXV") OR ("Nordic Society") OR ("NOPHO") OR ("thiopurine*") OR ("Antifolate*") OR ("Vinca Alkaloid*") OR ("Alkylating agent*") OR ("Alkylator*") OR ("Pyrimidine*") OR INDEXTERMS ("Antineoplastic Agents, Alkylating") OR INDEXTERMS ("Antimetabolites, Antineoplastic") OR INDEXTERMS ("Antibiotics, Antineoplastic") AND ("Genetic polymorphism*") OR ("Single nucleotide polymorphism*") OR ("Genetic variant*") OR ("Pharmacogen*") OR ("Mutation*") OR ("Personalized Medicine") OR ("Polymorphism*") OR (

"Variant*") OR ("Precision medicine") OR ("germline mutation") OR ("genetic association study") OR ("genotype") OR ("genetic background") OR ("genotyping technique") OR ("genetic heterogeneity") OR ("genetic profile") OR INDEXTERMS ("Polymorphism, Genetic") OR INDEXTERMS ("Pharmacogenomic Variants") OR INDEXTERMS ("Polymorphism, Single Nucleotide") OR INDEXTERMS ("Pharmacogenetics") OR INDEXTERMS ("Genome-Wide Association Study") OR INDEXTERMS ("Genomics") OR INDEXTERMS ("Germ-Line Mutation") OR INDEXTERMS ("Mutation") OR INDEXTERMS ("Precision Medicine") OR INDEXTERMS ("Genetic Association Studies") OR INDEXTERMS ("Genotype") OR INDEXTERMS ("Alleles") AND ("Drug-Related Side Effects" OR INDEXTERMS ("Adverse Reactions") OR INDEXTERMS ("Heart failure") OR "Chemical" OR INDEXTERMS ("Drug Induced Liver Injury") OR INDEXTERMS ("Chemotherapy-Related Cognitive Impairment") OR INDEXTERMS ("Drug Hypersensitivity") OR INDEXTERMS ("Leukopenia") OR INDEXTERMS ("Agranulocytosis") OR INDEXTERMS ("Neutropenia") OR INDEXTERMS ("Febrile Neutropenia") OR INDEXTERMS ("Chemotherapy-Induced Febrile Neutropenia") OR INDEXTERMS ("Anemia") OR INDEXTERMS ("Thrombocytopenia") OR INDEXTERMS ("Polyneuropathy") OR INDEXTERMS ("Thromboembolism") OR INDEXTERMS ("Embolism, Paradoxical") OR "Intracranial Embolism" OR INDEXTERMS ("Thrombosis") OR INDEXTERMS ("Carotid Artery Thrombosis") OR INDEXTERMS ("Intracranial Embolism") OR INDEXTERMS ("Intracranial Thrombosis") OR INDEXTERMS ("Venous Thromboembolism") OR INDEXTERMS ("Pancreatitis") OR INDEXTERMS ("Amylase") OR INDEXTERMS ("Lipase") OR INDEXTERMS ("Hyperglycemia") OR INDEXTERMS ("Hepatic Insufficiency") OR INDEXTERMS ("Liver Failure") OR INDEXTERMS ("End Stage Liver Disease") OR INDEXTERMS ("Hepatic Encephalopathy") OR INDEXTERMS ("Liver Failure, Acute") OR INDEXTERMS ("Acute kidney injury") OR INDEXTERMS ("Treatment outcome") OR INDEXTERMS ("Survival Rate") OR INDEXTERMS ("Overall survival") OR INDEXTERMS ("Disease-Free Survival") OR ("Adverse drug reaction*") OR ("Chemotherapy related toxicit*") OR ("Drug toxicit*") OR ("Adverse effect*") OR ("Toxicit*") OR ("Peripheral neuropath*") OR ("Neuropath*") OR ("Polyneuropathy") OR ("Myelosuppression") OR ("Hypersensitivit*") OR ("Allergic reaction*") OR ("Osteonecrosis") OR ("Hepatotoxicity") OR ("Nephrotoxicity") OR ("Heart failure") OR ("Drug Hypersensitivit*") OR ("Leukopenia") OR ("Agranulocytosis") OR ("Neutropenia") OR ("Febrile Neutropenia") OR ("Chemotherapy-Induced Febrile Neutropenia") OR ("Anemia") OR ("Thrombocytopenia") OR ("Thrombosis") OR ("Thromboembolism") OR ("Thromboembolic event*") OR ("Intracranial Embolism Thrombosis") OR ("Carotid Artery Thrombosis") OR ("Intracranial Embolism") OR ("Intracranial Thrombosis") OR ("Venous Thromboembolism") OR ("Pancreatitis") OR ("Acute pancreatitis") OR ("Amylase") OR ("Lipase") OR ("Hyperglycemia") OR ("Drug induced diabetes") OR ("Hepatic Insufficiency") OR ("Liver Failure") OR ("End Stage Liver Disease") OR ("Hepatic Encephalopathy") OR ("Acute liver failure") OR ("Acute kidney injury") OR ("Treatment outcome") OR ("Survival Rate") OR ("Overall survival") OR ("Disease-Free Survival") OR ("Leukemia free survival") OR ("Relapse free survival")) AND (EXCLUDE (SRCTYPE , "k") OR EXCLUDE (SRCTYPE , "b") OR EXCLUDE (SRCTYPE , "d")) AND (EXCLUDE (SUBJAREA

, "AGRI") OR EXCLUDE (SUBJAREA , "ARTS") OR EXCLUDE (SUBJAREA , "BUSI") OR EXCLUDE (SUBJAREA , "CENG") OR EXCLUDE (SUBJAREA , "CHEM") OR EXCLUDE (SUBJAREA , "COMP") OR EXCLUDE (SUBJAREA , "DENT") OR EXCLUDE (SUBJAREA , "EART") OR EXCLUDE (SUBJAREA , "MEDI") OR EXCLUDE (SUBJAREA , "BIOC") OR EXCLUDE (SUBJAREA , "IMMU") OR EXCLUDE (SUBJAREA , "ENVI") OR EXCLUDE (SUBJAREA , "NEUR") OR EXCLUDE (SUBJAREA , "MULT") OR EXCLUDE (SUBJAREA , "ENGI") OR EXCLUDE (SUBJAREA , "MATE") OR EXCLUDE (SUBJAREA , "PHYS") OR EXCLUDE (SUBJAREA , "MATH") OR EXCLUDE (SUBJAREA , "VETE") OR EXCLUDE (SUBJAREA , "SOCI") OR EXCLUDE (SUBJAREA , "PSYC") OR EXCLUDE (SUBJAREA , "ENER") OR EXCLUDE (SUBJAREA , "ECON") OR EXCLUDE (SUBJAREA , "DECI")) AND (LIMIT-TO (PUBYEAR , 2021) OR LIMIT-TO (PUBYEAR , 2020) OR LIMIT-TO (PUBYEAR , 2019) OR LIMIT-TO (PUBYEAR , 2018) OR LIMIT-TO (PUBYEAR , 2017) OR LIMIT-TO (PUBYEAR , 2016) OR LIMIT-TO (PUBYEAR , 2015) OR LIMIT-TO (PUBYEAR , 2014) OR LIMIT-TO (PUBYEAR , 2013) OR LIMIT-TO (PUBYEAR , 2012) OR LIMIT-TO (PUBYEAR , 2011) OR LIMIT-TO (PUBYEAR , 2010) OR LIMIT-TO (PUBYEAR , 2009) OR LIMIT-TO (PUBYEAR , 2008) OR LIMIT-TO (PUBYEAR , 2007) OR LIMIT-TO (PUBYEAR , 2006) OR LIMIT-TO (PUBYEAR , 2005) OR LIMIT-TO (PUBYEAR , 2004) OR LIMIT-TO (PUBYEAR , 2003) OR LIMIT-TO (PUBYEAR , 2002) OR LIMIT-TO (PUBYEAR , 2001) OR LIMIT-TO (PUBYEAR , 2000) OR LIMIT-TO (PUBYEAR , 1999) OR LIMIT-TO (PUBYEAR , 1998) OR LIMIT-TO (PUBYEAR , 1997) OR LIMIT-TO (PUBYEAR , 1996) OR LIMIT-TO (PUBYEAR , 1995)) AND (LIMIT-TO (LANGUAGE , "English")) AND (EXCLUDE (EXACTKEYWORD , "Nonhuman") OR EXCLUDE (EXACTKEYWORD , "Animals") OR EXCLUDE (EXACTKEYWORD , "Cell Line, Tumor") OR EXCLUDE (EXACTKEYWORD , "IC50") OR EXCLUDE (EXACTKEYWORD , "Review") OR EXCLUDE (EXACTKEYWORD , "Chronic Myeloid Leukemia") OR EXCLUDE (EXACTKEYWORD , "Lymphoma") OR EXCLUDE (EXACTKEYWORD , "Nonhodgkin Lymphoma") OR EXCLUDE (EXACTKEYWORD , "Protein Bcl 2") OR EXCLUDE (EXACTKEYWORD , "Protein P53") OR EXCLUDE (EXACTKEYWORD , "Aged, 80 And Over"))

Web of science:

((TS=("Acute lymphoblastic leukemia" OR "acute lymphocytic leukemia" OR "acute leukemia" OR "acute lymphoid leukemia" OR "childhood leukemia")

AND

TS=("Vincristine" OR "Anthracycline*" OR "Daunorubicin" OR "Doxorubicin" OR "Mitoxantrone" OR "6-Mercaptopurine" OR "Methotrexate" OR "Asparaginase" OR "Glucocorticoid*" OR "Dexamethasone" OR "Prednisolone" OR "Steroid*" OR "Cyclophosphamide" OR "Cytarabine" OR "Induction Chemotherapy" OR "Maintenance Chemotherapy" OR "Consolidation Chemotherapy" OR "Mercaptopurine" OR "Berlin-

Frankfurt-Münster" OR "BFM" OR "Children's Oncology Group" OR "COG" OR "AALL" OR "GRAALL" OR "CALGB" OR "Dutch Childhood Oncology Group" OR "DCOG" OR "Dana Farber Cancer Institute Consortium" OR "DFCI" OR "United Kingdom Acute Lymphoblastic Leukaemia" OR "UKALL" OR "MRC UK" OR "St Jude" OR "TOTXV" OR "Nordic Society" OR "NOPHO" OR "thiopurine*" OR "Antifolate*" OR "Vinca Alkaloid*" OR "Alkylating agent*" OR "Alkylator*" OR "Pyrimidine*" OR "Antimetabolite*")

AND

TS=("Genetic polymorphism*" OR "Single nucleotide polymorphism*" OR "Genetic variant*" OR "Pharmacogen*" OR "Mutation*" OR "Personalized Medicine" OR "Polymorphism*" OR "Variant*" OR "Precision medicine" OR "germline mutation" OR "genetic association study" OR "genotype" OR "genetic background" OR "genotyping technique" OR "genetic heterogeneity" OR "genetic profile" OR "Genome-Wide Association Study" OR "Genomics" OR "Germ-Line Mutation" OR "Allele*")

AND

TS=("Adverse drug reaction*" OR "Chemotherapy related toxicit*" OR "Drug toxicit*" OR "Adverse effect*" OR "Toxicit*" OR "Peripheral neuropath*" OR "Neuropath*" OR "Polyneuropathy" OR "Myelosuppression" OR "Hypersensitivit*" OR "Allergic reaction*" OR "Osteonecrosis" OR "Hepatotoxicity" OR "Nephrotoxicity" OR "Heart failure" OR "Drug Hypersensitivit*" OR "Leukopenia" OR "Agranulocytosis" OR "Neutropenia" OR "Febrile Neutropenia" OR "Chemotherapy-Induced Febrile Neutropenia" OR "Anemia" OR "Thrombocytopenia" OR "Thrombosis" OR "Thromboembolism" OR "Thromboembolic event*" OR "Carotid Artery Thrombosis" OR "Intracranial Embolism" OR "Intracranial Thrombosis" OR "Venous Thromboembolism" OR "Pancreatitis" OR "Acute pancreatitis" OR "Amylase" OR "Lipase" OR "Hyperglycemia" OR "Drug induced diabetes" OR "Hepatic Insufficiency" OR "Liver Failure" OR "Chemical and Drug Induced Liver Injury" OR "Chemotherapy-Related Cognitive Impairment" OR "Hepatic Encephalopathy" OR "End Stage Liver Disease" OR "Hepatic Encephalopathy" OR "Acute liver failure" OR "Acute kidney injury" OR "Treatment outcome" OR "Survival Rate" OR "Overall survival" OR "Disease-Free Survival" OR "Leukemia free survival" OR "Relapse free survival"))

NOT

(TS=("Bone Marrow Transplant*" OR "Stem Cell Transplant*" OR "Stem Cell Mobilization" OR "Allograft*" OR "Autograft*" OR "Large granular lymphocytic leukemia" OR "Prolymphocytic leukemia" OR "adult leukemia" OR "chronic leukemia" OR "acute myeloid leukemia")))

AND LANGUAGE: (English)

Refined by: DOCUMENT TYPES: (ARTICLE)

AND PUBLICATION YEARS: (2021 OR 2013 OR 2005 OR 1998 OR 2020 OR 2012 OR 2004 OR 1997 OR 2019 OR 2011 OR 2003 OR 1996 OR 2018 OR 2010 OR 2002 OR 1995 OR 2017 OR 2009 OR 2001 OR 2016 OR 2008 OR 2000 OR 2015 OR 2007 OR 1999 OR 2014 OR 2006)

Supplementary Material S2: Link for CADIMA software :

https://www.cadima.info/index.php/area/generateDocx/1857

Supplementary Material S3: Data extraction template

2021_05_17_data_ex
traction_sheet.xlsx

Supplementary Material S4: Quality assessment using the STrengthening the Reporting of Pharmacogenetic Studies (STROPS) checklist

Quality analysis
scores for all studies

Consensus process for STROPS adaptation

The adaptation of the STROPS checklist was performed in three rounds:

- **Round 1:** Five reviewers (CRSU, SG, VV, SS, SC) independently reviewed all 54 items and marked those they considered essential for evaluating internal validity and reporting transparency in pharmacogenetic association studies.
- **Round 2:** Items marked as essential by at least 3 of 5 reviewers were retained (n = 28). Items marked by 0–2 reviewers were discussed.
- **Round 3:** A group discussion was held to resolve disagreements. Five items were added back after discussion, and 10 were excluded as redundant or not directly relevant to bias assessment. The final set of 23 items was agreed unanimously.

The final retained items assess: participant selection and characterisation, genotyping validity, outcome definitions, statistical analyses (including adjustment for population stratification and multiple testing), and transparent reporting of results (genotype frequencies, HWE, genetic models). Excluded items primarily related to broader study context (e.g., funding sources, general introductions, ethical approval, discussion sections) or duplicated other retained items.

Supplementary Material S5: List of Genes and Genetic Variants (with rs IDs)

list of Gnes and Genetic variants .xls

Supplementary Table S1. Detailed characteristics of the pharmacogenetic studies included in the systematic review evaluating treatment-related toxicities in pediatric acute lymphoblastic leukemia (n = 68).

Study	Author	Year	Country	Study design	Gene / Variant investigated	Treatment-related toxicity (TRT)
1	Choi R et al.	2019	Korea	Cohort	Thiopurine pathway genes	Myelosuppression
2	Ansari M et al.	2012	Canada	Cohort	MRP3	Chemotherapy toxicity
3	French D et al.	2008	USA	Cohort	SERPINE1	Osteonecrosis
4	Ramsey LB et al.	2017	USA	Cohort	Dexamethasone pathway genes	Glucocorticoid toxicity
5	Gagné V et al.	2019	Canada	GWAS	ACP1	Osteonecrosis
6	Aplenc R et al.	2003	USA	Case-control	CYP3A	Treatment toxicity
7	Yanagimachi M et al.	2013	Japan	Cohort	MTHFR	Methotrexate toxicity
8	Razali RH et al.	2020	Malaysia	Cohort	ABCC2	Methotrexate toxicity
9	Grimes AC et al.	2021	USA	Cohort	CFTR	Pancreatitis
10	Kim H et al.	2018	Korea	Cohort	APEX1	Neutropenia
11	Erčulj N et al.	2012	Slovenia	Cohort	Folate pathway genes	Methotrexate toxicity
12	Lund B et al.	2014	Denmark	Cohort	Host genome variants	Infection
13	Zhou Y et al.	2020	China	Cohort	TPMT, NUDT15	Myelosuppression
14	Abaji R et al.	2018	Canada	Exome sequencing	Multiple genes	Neuropathy
15	Wolthers BO et al.	2017	Denmark	Cohort	ULK2	Pancreatitis
16	Relling MV et al.	2004	USA	Cohort	Pharmacogenetic variants	Osteonecrosis
17	Kutszegi N et al.	2015	Hungary	Cohort	HLA	Asparaginase hypersensitivity
18	Cheng Y et al.	2021	China	Cohort	SLCO1B1	Methotrexate clearance

19	Moulik NR et al.	2016	India	Cohort	MTHFR	Methotrexate toxicity
20	Kahn JM et al.	2018	USA	Cohort	Pharmacogenetic variants	Toxicity
21	Yousef AM et al.	2019	Jordan	Cohort	Folate pathway genes	Methotrexate toxicity
22	Gervasini G et al.	2017	Spain	Cohort	Transporter genes	Chemotherapy toxicity
23	Li L et al.	2019	USA	Cohort	CEP72	Vincristine neuropathy
24	Zaman S et al.	2019	Bangladesh	Cohort	TPMT, ITPA	Hematologic toxicity
25	Højfeldt SG et al.	2019	Denmark	Cohort	HLA	Asparaginase hypersensitivity
26	Diouf B et al.	2015	USA	Cohort	CEP72	Peripheral neuropathy
27	Kawedia JD et al.	2011	USA	Cohort	Multiple genes	Osteonecrosis
28	Karol SE et al.	2016	USA	Cohort	Genetic variants	Osteonecrosis
29	Roy Moulik N et al.	2015	India	Cohort	MTHFR	Methotrexate toxicity
30	Kaymak Cihan M et al.	2017	Turkey	Cohort	NR3C1	Steroid toxicity
31	Abaji R et al.	2017	Canada	Exome sequencing	Asparaginase genes	Pancreatitis
32	Liu SG et al.	2011	China	Cohort	MTHFR	Methotrexate toxicity
33	Gregers J et al.	2015	Denmark	Cohort	ABCB1	Chemotherapy toxicity
34	Karas-Kuželički N et al.	2016	Slovenia	Cohort	Risk variants	Osteonecrosis
35	Kutszegi N et al.	2021	Hungary	Cohort	HLA	Hypersensitivity
36	Erdélyi DJ et al.	2006	Hungary	Cohort	ABC transporters	Immunosuppression
37	Abo-Bakr A et al.	2017	Egypt	Cohort	XRCC1	Chemotherapy toxicity
38	ElFayoumi RI et al.	2019	Saudi Arabia	Cohort	ABCB1	Glucocorticoid toxicity
39	Haase R et al.	2012	Germany	Cohort	MTHFR	Methotrexate toxicity
40	Ansari M et al.	2009	Switzerland	Cohort	MRP4	Treatment outcome
41	Ceppi F et al.	2014	Canada	Cohort	Vincristine pathway genes	Neuropathy
42	Moriyama T et al.	2016	USA	Cohort	NUDT15	Myelosuppression
43	Yang JJ et al.	2015	USA	Cohort	NUDT15	Mercaptopurine toxicity
44	Soler AM et al.	2018	Uruguay	Cohort	TPMT, NUDT15	Myelosuppression
45	Linga VG et al.	2014	India	Cohort	TPMT	Hematologic toxicity
46	Yi ES et al.	2018	Korea	Cohort	NUDT15	Hematologic toxicity
47	Chae H et al.	2020	Korea	Cohort	MTHFR	Methotrexate toxicity
48	Pakakasama S et al.	2007	Thailand	Cohort	Folate pathway genes	Methotrexate toxicity
49	Yazıcıoğlu B et al.	2017	Turkey	Cohort	Folate pathway genes	Methotrexate toxicity
50	Al-Sheikh A et al.	2021	Jordan	Cohort	TYMS	Methotrexate toxicity
51	Kim H et al.	2012	Korea	Cohort	ITPA	Hematologic toxicity
52	Liu Y et al.	2014	China	Cohort	ABCC2	Methotrexate toxicity

53	Pakakasama S et al.	2007	Thailand	Cohort	Folate genes	MTX toxicity
54	Yousef AM et al.	2019	Jordan	Cohort	Folate genes	MTX toxicity
55	Haase R et al.	2012	Germany	Cohort	MTHFR	MTX toxicity
56	Cheng Y et al.	2021	China	Cohort	SLCO1B1	MTX clearance
57	Razali RH et al.	2020	Malaysia	Cohort	ABCC2	MTX toxicity
58	Chae H et al.	2020	Korea	Cohort	MTHFR	MTX toxicity
59	Liu SG et al.	2011	China	Cohort	MTHFR	MTX toxicity
60	Moulik NR et al.	2016	India	Cohort	MTHFR	MTX toxicity
61	Roy Moulik N et al.	2015	India	Cohort	MTHFR	MTX toxicity
62	Erculj N et al.	2012	Slovenia	Cohort	Folate pathway genes	MTX toxicity
63	Yanagimachi M et al.	2013	Japan	Cohort	MTHFR	MTX toxicity
64	Razali RH et al.	2020	Malaysia	Cohort	ABCC2	MTX toxicity
65	Liu Y et al.	2014	China	Cohort	ABCC2	MTX toxicity
66	Choi R et al.	2019	Korea	Cohort	NUDT15	Myelosuppression
67	Moriyama T et al.	2016	USA	Cohort	NUDT15	Myelosuppression
68	Yang JJ et al.	2015	USA	Cohort	NUDT15	Mercaptopurine toxicity

Supplementary Table S2. Quality assessment of the included studies using the adapted STROPS (Strengthening the Reporting of Pharmacogenetic Studies) checklist. Each item was scored as 1 (reported) or 0 (not reported) with a maximum score of 23 points per study. Studies scoring 16–23 were considered high quality, whereas scores 8–15 were considered moderate quality.

Study	Author	Year	STROPS score	Quality category
1	Liu Y et al.	2014	14	Moderate
2	Kim H et al.	2012	15	Moderate
3	Yazıcıoğlu B et al.	2017	13	Moderate
4	Al-Sheikh A et al.	2020	15	Moderate
5	Choi R et al.	2019	20	High
6	Ansari M et al.	2012	18	High
7	French D et al.	2008	19	High
8	Ramsey LB et al.	2017	19	High
9	Kishi S et al.	2003	14	Moderate
10	Gervasini G et al.	2017	14	Moderate
11	Gagné V et al.	2019	22	High
12	Kishi S et al.	2007	17	High
13	Aplenc R et al.	2003	16	High
14	Yanagimachi M et al.	2013	16	High
15	Razali RH et al.	2020	16	High
16	Grimes AC et al.	2021	12	Moderate

17	Kim H et al.	2018	13	Moderate
18	Erčulj N et al.	2012	21	High
19	Lund B et al.	2014	19	High
20	Zhou Y et al.	2020	15	Moderate
21	Abaji R et al.	2018	19	High
22	Gervasini G et al.	2017	16	High
23	Wolthers BO et al.	2017	21	High
24	Relling MV et al.	2004	17	High
25	Kutszegi N et al.	2020	16	High
26	Pakakasama S et al.	2007	13	Moderate
27	Kutszegi N et al.	2015	21	High
28	Cheng Y et al.	2021	22	High
29	Moulik NR et al.	2016	11	Moderate
30	Kahn JM et al.	2018	16	High
31	Yousef AM et al.	2019	14	Moderate
32	Faganel Kotnik B et al.	2010	9	Moderate
33	Soler AM et al.	2018	11	Moderate
34	Linga VG et al.	2014	12	Moderate
35	Burgueño-Rodríguez G et al.	2020	18	High
36	Hareedy MS et al.	2015	19	High
37	Ben Tanfous M et al.	2015	19	High
38	Xue Y et al.	2015	18	High
39	Li L et al.	2019	21	High
40	Karas-Kuželički N et al.	2016	19	High
41	Chae H et al.	2020	15	Moderate
42	Zaman S et al.	2019	17	High
43	Højfeldt SG et al.	2019	20	High
44	Yi ES et al.	2018	15	Moderate
45	Erdélyi DJ et al.	2006	15	Moderate
46	Eipel O et al.	2016	12	Moderate
47	Diouf B et al.	2015	19	High
48	Kawedia JD et al.	2011	19	High
49	Abo-Bakr A et al.	2017	15	Moderate
50	Gregers J et al.	2015	17	High
51	ElFayoumi RI et al.	2019	14	Moderate
52	Karol SE et al.	2016	20	High
53	Moulik NR et al.	2015	16	High
54	Kaymak Cihan M et al.	2017	17	High
55	Krajinovic M et al.	2017	21	High
56	Liu SG et al.	2011	17	High
57	Pakakasama S et al.	2007	10	Moderate
58	Tantawy AAG et al.	2010	17	High
59	Plesa M et al.	2019	18	High
60	Haase R et al.	2012	15	Moderate
61	Karol SE et al.	2015	22	High
62	Sepe DM et al.	2012	21	High
63	Ansari M et al.	2009	11	Moderate
64	Bernbeck B et al.	2003	13	Moderate

65	Cao M et al.	2020	16	High
66	Adam de Beaumais T et al.	2011	19	High
67	Liu SG et al.	2013	20	High
68	Ceppi F et al.	2014	15	Moderate

Table S3. Pearson correlation analysis of minor allele frequencies between Indigenome and gnomAD populations.

**Population comparison**	**Pearson's r**	**P-value**
Indigenome vs gnomAD (All populations, AF)	0.919	1.6×10^{-53}
Indigenome vs gnomAD South Asian (SAS)	0.997	6.4×10^{-141}
Indigenome vs gnomAD Non-Finnish European (NFE)	0.901	2.3×10^{-48}

Supplementary Table S4. Original research studies published after the primary search cutoff (post-10 January 2021) that are cited in the Discussion as emerging evidence supporting the review's findings

**First author (year) Reference .**	**Study design**	**Population**	**Genes/variants**	**Toxicity area**	**Key finding as cited in the Discussion**
Maillard M (2024) [101]	Multi-ethnic cohort	Children with ALL (multiethnic)	*TPMT*, *NUDT15*	Thiopurine-induced myelosuppression	Additive effects of *TPMT* and *NUDT15* variants: patients with reduced activity in both enzymes tolerate substantially lower mercaptopurine doses and exhibit increased hematopoietic toxicity compared with single-variant carriers
Khaeso K (2026) [102]	Prospective cohort	Thai children with ALL	*NUDT15*, *TPMT*	Thiopurine-induced hematopoietic toxicity	Genetic determinants of hematopoietic toxicity risk in Thai paediatric patients

					undergoing 6-mercaptopurine treatment
Goodenough CG (2022) [105]	Long-term survivor cohort	Survivors of childhood ALL	*CEP72*	Vincristine-induced peripheral neuropathy	Carriers of the *CEP72* risk allele have an increased risk of persistent motor neuropathy following vincristine exposure
Junk SV (2023)[109]	Genome-wide association study (GWAS)	Children with ALL (AIEOP-BFM cohort)	*UGT1A* cluster	Chemotherapy-related hyperbilirubinemia	Strong association between variants within the *UGT1A* gene cluster and hyperbilirubinemia; rs6744284 linked to *UGT1A1*28/*37* alleles impairing bilirubin glucuronidation
Jardim LC (2026) [112]	Retrospective observational cohort	Children with ALL/lymphoma	*ABCC2*, *ABCC4*, *ABCC1*, *MTHFR*	Oral mucositis	Variants in transporter and metabolic genes associated with increased risk of chemotherapy-induced oral mucositis
Zhang XYC (2026)[113]	Pathway-informed machine learning (original research)	Children with ALL	Polygenic models (inflammatory and mucosal pathway genes)	Methotrexate-induced mucositis	Polygenic models incorporating multiple inflammatory and mucosal pathway genes improve prediction of methotrexate-induced mucositis
Larkin T (2023)[114]	Single-center cohort	75 children with ALL	116 SNPs across drug pathways	Polygenic toxicity risk scores (gastrointestinal, neurological, endocrine, haematological)	Three-SNP models improved prediction of toxicities compared with single-SNP approaches

Supplementary Table S5 – Mapping of STROPS checklist items

Below is the complete table showing all 54 STROPS items with the retained items marked. The 23 retained items are exactly those that correspond to the columns in your scoring table.

#	STROPS Item	Retained?	Reason for exclusion (if not retained)
1	Provide in the abstract an informative and balanced summary	No	Abstract content is a reporting requirement, not a quality/validity assessment criterion
2	Explain the scientific background and rationale	No	Relates to general introduction, not genetic association validity
3	Provide reasons for choosing the genes and SNPs genotyped	**Yes**	Assesses appropriateness of genetic exposure selection
4	State specific objectives, including any prespecified hypotheses	**Yes**	Assesses clarity of study aims
5	State if the study is the first report or a replication effort	No	Relates to study context, not internal validity
6	Present key elements of study design early	**Yes**	Assesses clarity of study design
7	Describe setting, locations, and relevant dates	No	Relates to general study context, not genetic association validity
8	Give eligibility criteria and methods of participant selection	**Yes**	Assesses participant selection rigour
9	Report the drug and regime participants were exposed to	**Yes**	Assesses drug exposure clarity
10	For matched case-control study, give matching criteria	No	Specific to case-control studies; not applicable to all included studies
11	Give information on selection of subsets from larger study	No	Specific to subset analyses; not applicable to all studies
12	Provide information on patient cohort overlap with other publications	No	Relates to study context, not internal validity
13	Report disease/clinical indication using standardised ontology	**Yes**	Assesses clinical characterisation
14	Clearly define all outcomes, potential confounders, and effect modifiers	**Yes**	Assesses outcome definition and confounder identification
15	Provide justification for choice of outcomes	No	Relates to study rationale, not internal validity

16	Clearly define genetic exposures using widely used nomenclature	**Yes**	Assesses genetic exposure clarity
17	Report the rs number of each genotyped SNP	**Yes**	Assesses genetic exposure clarity
18	Clearly state how haplotypes or star alleles were defined	**Yes**	Assesses genetic exposure clarity
19	State which allele is minor/major/reference/risk and for which population	**Yes**	Assesses allele definition clarity
20	For each variable, give sources of data and methods of assessment	**Yes**	Assesses data collection rigour
21	Describe laboratory methods, genotyping platforms, error rates	**Yes**	Assesses genotyping validity
22	Describe genotype quality control methods and findings	**Yes**	Assesses genotyping validity
23	For quantitative outcomes, investigate potential bias from pharmacotherapy	No	Specific to quantitative outcomes; not applicable to all studies
24	Report how adherence to treatment was assessed	No	Relates to treatment compliance, not genetic association validity
25	Explain how study size was arrived at or power to detect effects	**Yes**	Assesses statistical power
26	Explain how quantitative variables were handled in analyses	**Yes**	Assesses analytical rigour
27(a)	Describe methods used to control for confounding	**Yes**	Assesses confounder adjustment
27(b)	Describe any methods used to examine subgroups and interactions	**Yes**	Assesses analytical rigour
27(c)	Explain how missing data were addressed	**Yes**	Assesses analytical rigour
27(d)	Explain how loss to follow-up was addressed	No	Specific to cohort studies; not applicable to all included studies
27(e)	Explain how matching of cases and controls was addressed	No	Specific to case-control studies; not applicable to all included studies
27(f)	Describe any sensitivity analyses	**Yes**	Assesses analytical rigour
28	State whether Hardy–Weinberg equilibrium was considered	**Yes**	Assesses genetic data quality

29	Describe any methods used for inferring genotypes or haplotypes	No	Not applicable if not performed; duplicates item 18
30	Describe any methods used to assess or address population stratification	**Yes**	Assesses population stratification control
31	Describe any methods used to assess and correct for relatedness	No	Not applicable to all studies
32(a–c)	Describe methods to address multiple comparisons/ false positives	**Yes**	Assesses multiple testing correction
33	Describe methods to adjust for extent of adherence	No	Relates to treatment adherence, not genetic association validity
34	Report numbers of individuals at each stage of the study	**Yes**	Assesses participant flow and sample size reporting
35	Report any SNPs that were excluded and reasons	No	Duplicates item 22; not applicable to all studies
36	Give characteristics of study participants and potential confounders	**Yes**	Assesses participant characterisation
37	Cohort study—Summarize follow-up time	No	Specific to cohort studies; not applicable to all included studies
38	Highlight SNPs that deviate from HWE	No	Duplicates item 28
39	Where population stratification is assessed, report results	No	Duplicates item 30
40(a–c)	Report outcomes for each genotype category	**Yes**	Assesses outcome reporting by genotype
41	If more than one ethnic group, provide summary data per ethnic group	No	Not applicable to all studies; duplicates item 36
42	Give unadjusted and adjusted estimates with precision	**Yes**	Assesses effect estimate reporting
43	Report category boundaries for continuous variables	No	Specific to certain analyses; not applicable to all studies
44	Report other analyses—subgroups, interactions, sensitivity	No	Duplicates items 27(b) and 27(f)
45	If numerous genetic variants examined, summarise results from all analyses	**Yes**	Assesses completeness of results reporting
46	If detailed results are in supplementary materials, state how to access	No	Relates to supplementary access, not internal validity

47	Summarize key results with reference to study objectives	No	Relates to discussion, not genetic association validity
48	Discuss limitations of the study	No	Relates to discussion, not genetic association validity
49	Give cautious overall interpretation of results	No	Relates to discussion, not genetic association validity
50	Discuss generalisability (external validity) of results	No	Relates to discussion, not genetic association validity
51	State whether the study has been registered	No	Relates to study context, not internal validity
52	Report whether ethical approval was obtained	No	Relates to study context, not internal validity
53	Give source of funding and role of funders	No	Relates to study context, not internal validity
54	State whether databases are or will become publicly available	No	Relates to study context, not internal validity

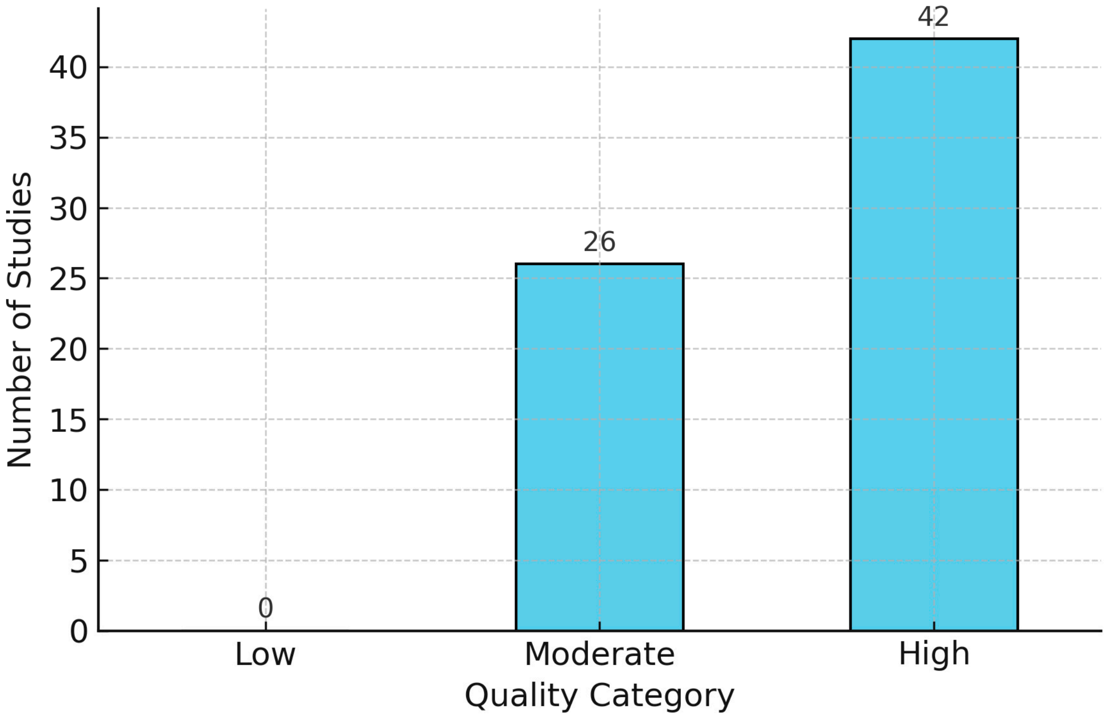

Figure S1. Distribution of STROPS quality assessment scores across the included studies.

The figure summarises the overall quality scores obtained using the adapted 23-item STROPS checklist. Studies were categorised as high quality (scores 16–23) or moderate quality (scores 8–15), and the distribution of scores across the included studies is presented.

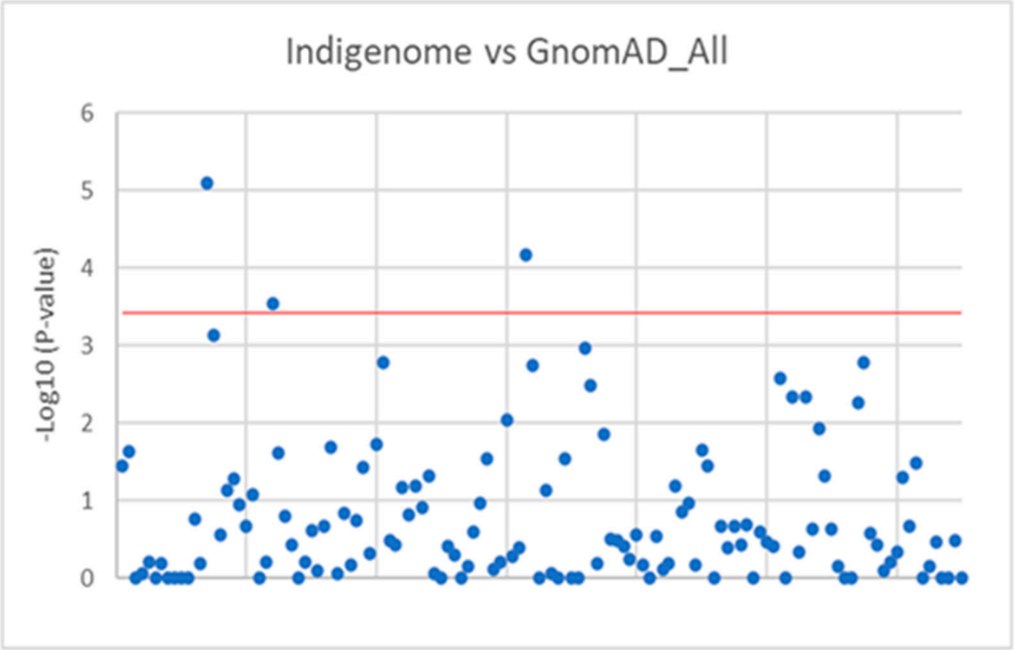

Figure S2. Fisher's exact test analysis comparing minor allele frequencies between the IndiGenomes dataset and gnomAD populations.

Each point represents a pharmacogenetic variant evaluated in the allele frequency comparison analysis. The y-axis shows the −log10-transformed P values derived from Fisher's exact test. The horizontal threshold line indicates statistical significance.

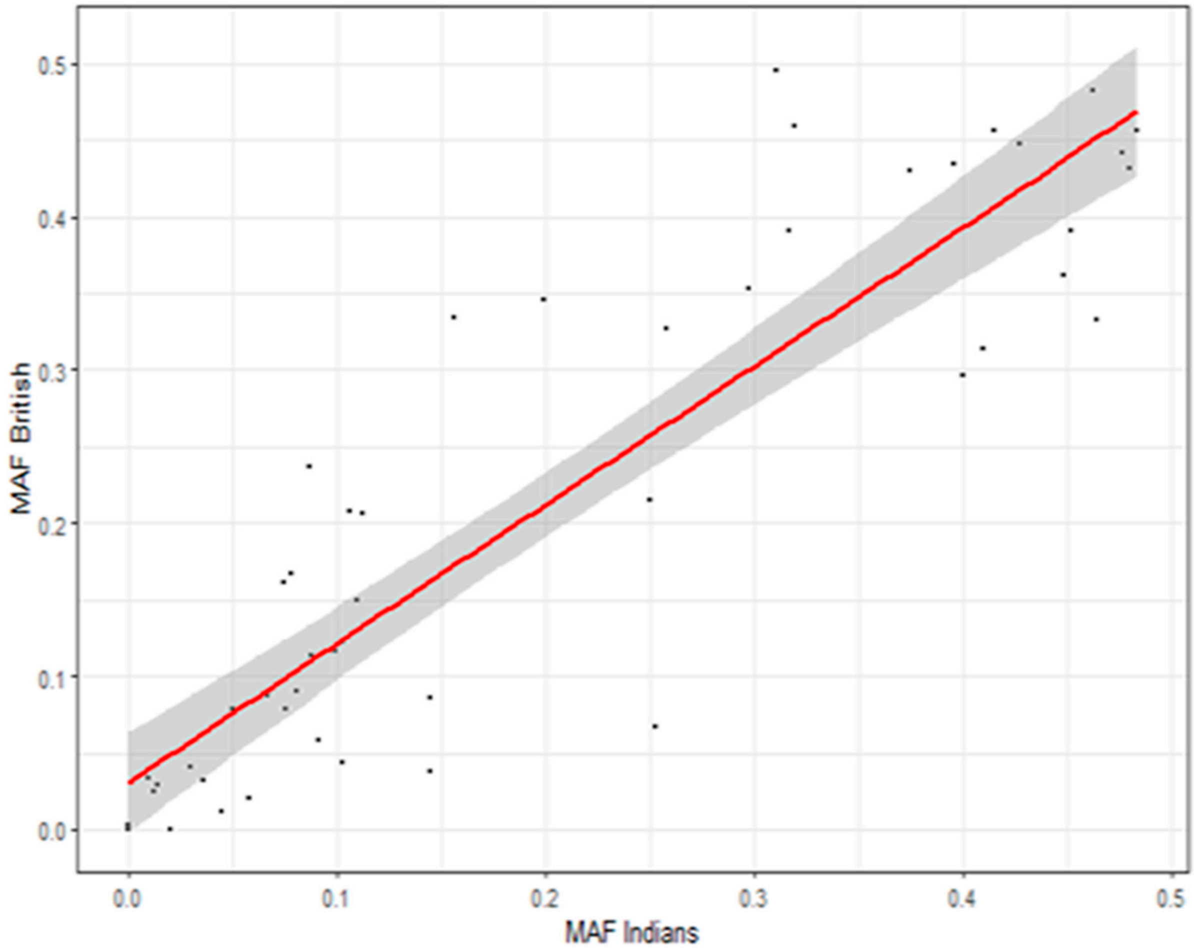

Figure S3. Comparison of allele frequencies between the UK Biobank Indian ancestry group and the British population. This research has been conducted using summary data from the UK Biobank Resource (https://www.ukbiobank.ac.uk/). We thank the participants and the UK Biobank team for their contributions.

Scatter plots illustrate the relationship between the allele frequencies observed in individuals of Indian ancestry and those in the UK Biobank British population. The regression line represents the overall correlation between the two populations.
